# Intrinsic host susceptibility among multiple species to intranasal SARS-CoV-2 identifies diverse virological, biodistribution and pathological outcomes

**DOI:** 10.1038/s41598-022-23339-x

**Published:** 2022-11-04

**Authors:** Neil Berry, Deborah Ferguson, Sarah Kempster, Jo Hall, Claire Ham, Adrian Jenkins, Vicky Rannow, Elaine Giles, Rose Leahy, Sara Goulding, Arturo Fernandez, Yemisi Adedeji, Sandrine Vessillier, Deepa Rajagopal, Sandra Prior, Yann Le Duff, Matthew Hurley, Sarah Gilbert, Martin Fritzsche, Ryan Mate, Nicola Rose, Robert J. Francis, Kirsty MacLellan-Gibson, Alejandro Suarez-Bonnet, Simon Priestnall, Neil Almond

**Affiliations:** 1 Division of Infectious Disease Diagnostics, NIBSC, Hertfordshire, UK; 2 Division of Analytical and Biological Sciences, NIBSC, Hertfordshire, UK; 3 Division of Virology, NIBSC, Hertfordshire, UK; 4 Division of Biotherapeutics, NIBSC, Hertfordshire, UK; 5 Department of Pathobiology and Population Sciences, The Royal Veterinary College, Hertfordshire, UK

**Keywords:** Diseases, Pathogenesis

## Abstract

SARS-CoV-2 exhibits a diverse host species range with variable outcomes, enabling differential host susceptibility studies to assess suitability for pre-clinical countermeasure and pathogenesis studies. Baseline virological, molecular and pathological outcomes were determined among multiple species—one Old World non-human primate (NHP) species (cynomolgus macaques), two New World NHP species (red-bellied tamarins; common marmosets) and Syrian hamsters—following single-dose, atraumatic intranasal administration of SARS-CoV-2/Victoria-01. After serial sacrifice 2, 10 and 28-days post-infection (dpi), hamsters and cynomolgus macaques displayed differential virus biodistribution across respiratory, gastrointestinal and cardiovascular systems. Uniquely, New World tamarins, unlike marmosets, exhibited high levels of acute upper airway infection, infectious virus recovery associated with mild lung pathology representing a host previously unrecognized as susceptible to SARS-CoV-2. Across all species, lung pathology was identified post-clearance of virus shedding (antigen/RNA), with an association of virus particles within replication organelles in lung sections analysed by electron microscopy. Disrupted cell ultrastructure and lung architecture, including abnormal morphology of mitochondria 10–28 dpi, represented on-going pathophysiological consequences of SARS-CoV-2 in predominantly asymptomatic hosts. Infection kinetics and host pathology comparators using standardized methodologies enables model selection to bridge differential outcomes within upper and lower respiratory tracts and elucidate longer-term consequences of asymptomatic SARS-CoV-2 infection.

## Introduction

Emergence of pandemic SARS-CoV-2 has resulted in devasting effects on human health with widespread economic, social and public health consequences. SARS-CoV-2 is thought to have its origins in horseshoe bats having entered human populations as a novel zoonotic pathogen^1^. Identification and development of appropriate animal models accelerates development of effective interventions that either prevent or ameliorate infection and disease with new and emerging pathogens. A number of laboratory animal species may be infected with SARS-CoV-2, including Old World non-human primates (NHP), ferrets, cats, hamsters and mice expressing the viral receptor^2^. In such studies, it is important to understand whether observed differences in outcomes are attributable to intrinsic susceptibility of the laboratory animal species or experimental differences between the isolate, dose and methods of administering virus.

It is recognized no single model or species re-capitulates the full disease spectrum of human SARS-CoV-2 infection/Covid-19, where significant co-morbidities impact on outcome severity^3^. However, careful selection of a model that recapitulates a particular aspect of SARS-CoV-2 pathogenesis, may enable specific questions to be addressed leading to a greater understanding of pathological processes. Continued appearance of novel variants of concern^4^ with differing biological properties further underscores the public health requirement for continued evaluation of in vivo infectivity, virulence, immunogenicity, cross-protection and pathology outcomes using suitable model systems.

The primary cellular receptor for SARS-CoV-2, angiotensin I converting enzyme-2 (ACE-2) is widely represented among vertebrate species accounting for the potentially very wide host range of SARS-CoV-2^5^, mediating binding to the viral spike (S)/receptor binding domain (RBD) with a broad tropism for SARS-CoV-2 S to mammalian ACE-2 proteins^6–9^, although the virus is capable of utilizing secondary receptors (e.g. TMPRSS2). Old World (Catarrhine) NHP species with 100% homology to human ACE-2 appear fully permissive for SARS-CoV-2^10–16^, mice displaying resistance to early pandemic SARS-CoV-2 strains with a diverse range of other species existing between these two extremes. Notably, early studies identified Syrian golden hamsters to be highly permissive for SARS-CoV-2, emerging as an important in vivo model to evaluate vaccines, therapeutics and pathogenesis^17–23^.

Previously, we performed a comparison of Zika virus infection in Old World (macaques) and New World (marmosets and tamarins) NHPs with a single well-characterised challenge stock^24^, to inform model selection for subsequent immune protection studies^25^. We applied the same principle to identify and select suitable models for equivalent studies for SARS-CoV-2. Understanding parameters of virus and host interactions is important in differential model selection for pathogenesis and medical countermeasure studies. Hence we sought to apply these principles to SARS-CoV-2. New World NHP species have also been relatively understudied.

Here, we identify viral kinetics and pathological consequences following atraumatic intranasal (I.N.)-only administration of a high dose, genetically well-characterised stock of the SARS-CoV-2/Victoria-01 strain isolated early in the pandemic^26^ in one Old World (*Macaca fascicularis*, cynomolgus macaque), two New World NHP species (*Callithrix jacchus*, common marmoset; *Saquinus labiatus*, red-bellied tamarin) and one small animal (*Mesocricetus auratus,* Syrian golden hamster). Serial sacrifice after challenge enabled a detailed analysis of the comparative viral distribution and pathology at light microscope and ultrastructural levels when replication in the upper respiratory tract was at its peak (day 2) and early (day 10) and late (day 28) times after viral suppression. Interspecies differences in infection dynamics and lung pathology identified red-bellied tamarins to be susceptible to infection with SARS-CoV-2, hitherto unrecognized, with outcomes across all species highlighting early structural changes in lung architecture and pathological outcomes in a species-independent manner. Identification of alterations in lung ultrastructure provide further insight into the prolonged impact of SARS-CoV-2 in predominantly asymptomatic hosts.

## Results

### Study outline and virus stock

**Figure 1:**
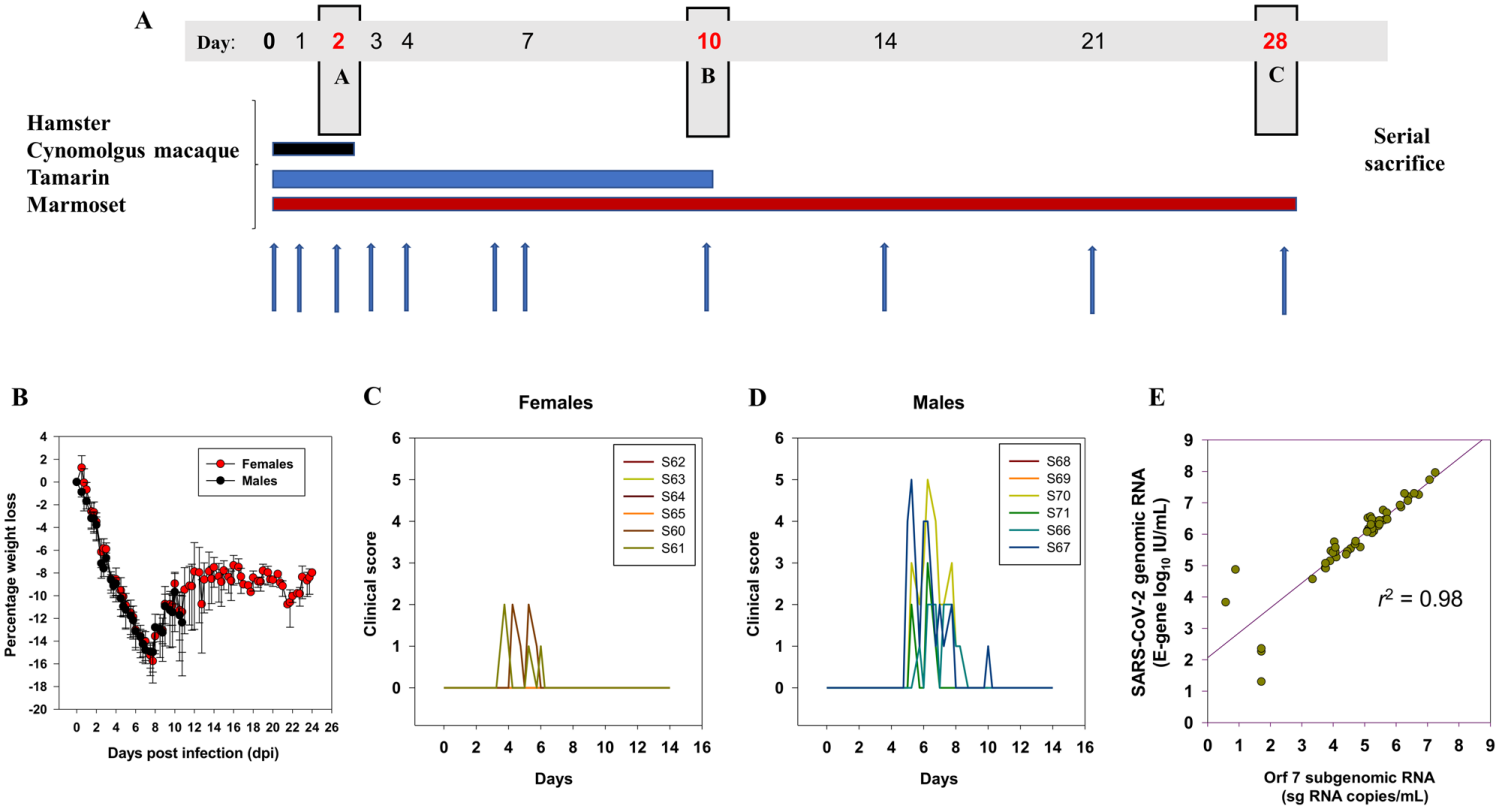
Study plan parameters. (**A**) Study outline for evaluation of SARS-CoV-2 in multiple species**.** Major termination time-points of A, B and C at 2, 10 and 28 days post-infection (dpi) respectively; vertical blue arrows indicate sampling frequencies. At termination, tissues taken for analysis were lung, trachea, oesophagus, olfactory bulb, tongue, tonsil, salivary gland LN, salivary gland, aorta, heart, kidney, liver, MLN, PLN-LN, spleen, rectum, small intestine, large intestine. (**B**) weight loss profiles in female and male hamsters as percentage weight change from baseline (day of challenge) over 28 days (females, red) and 11 days (males, black). SE bars indicated; (**C**, **D**) clinical score comparison between female and male hamsters respectively; (**E**) strong linear relationship between SARS-CoV-2 E-gene genomic RNA (gRNA) expressed as log_10_ IU/mL against subgenomic (sg) Orf7 RNA levels expressed as log_10_ copies/mL in 51 combined nasal and oral swabs over the first 10 days of Vic-01 infection.

A common study outline with serial sacrifice time-points at 2, 10 and 28 days post-infection (dpi) was used to compare virus replication and host pathology in each species, encompassing the acute (A), post-acute (B) and chronic phase (C) of SARS-CoV-2 infection and disease (Fig. 1A). ACE-2 sequence homology across the RBD for each study species are summarised in Fig. S1. A single common bulk virus stock was used in all challenges, the Victoria strain prepared after 4 passages on Vero/hSLAM cells (final concentration 2 × 10^6^ TCID_50_/mL on VeroE6/TMPRSS2 cells). Next generation sequencing determined this stock to be free of attenuating mutations reported to arise from in vitro propagation. No significant variants were called above a 1% threshold with respect to the Wuhan-01 reference sequence (Fig. S2). A single final dose containing 5 × 10^4^ TCID_50_ was administered atraumatically via the intranasal (I.N.) route to each of 12 Syrian golden hamsters (6 female, 6 male); 6 cynomolgus macaques (female) 4 common marmosets (2 female, 2 male) and 9 red-bellied tamarins (8 female, 1 male,). The virus was administered in a final volume of 50 µL for hamsters and New World monkeys and 500 µL for macaques.

### Clinical measures across species

Intranasal challenge in female and male hamsters resulted in significant weight loss to 7-8dpi, which approached but never exceeded 20% baseline weight (Fig. 1B), stabilising and returning towards pre-challenge levels. Mild clinical changes 4–7 dpi based upon a range of clinical scoring markers (Table S1) were higher in males than females peaking around the nadir of weight loss (Fig. 1C,D). Moderate hypo- and hyperthermic temperature spikes beyond the normal diurnal range 1–6 dpi were more marked in male hamsters (Fig. S3A). By contrast, all three NHP species remained clinically asymptomatic with no abnormal clinical signs recorded. Body temperature did not vary significantly from the normal diurnal temperature rhythm within each NHP species (Fig. S3B–D), except transient disturbance in cynomolgus macaques during the first 24 h post virus administration and a transient drop in temperature in tamarins at 24–48 h p.i. In cynomolgus macaques, blood haematology remained within the normal range over the first 10 days with only neutrophils elevated from 3 dpi. (Fig. S4).

### Comparative shedding of SARS-CoV-2/Vic-01 in Syrian hamsters and NHPs following intranasal challenge

**Figure 2:**
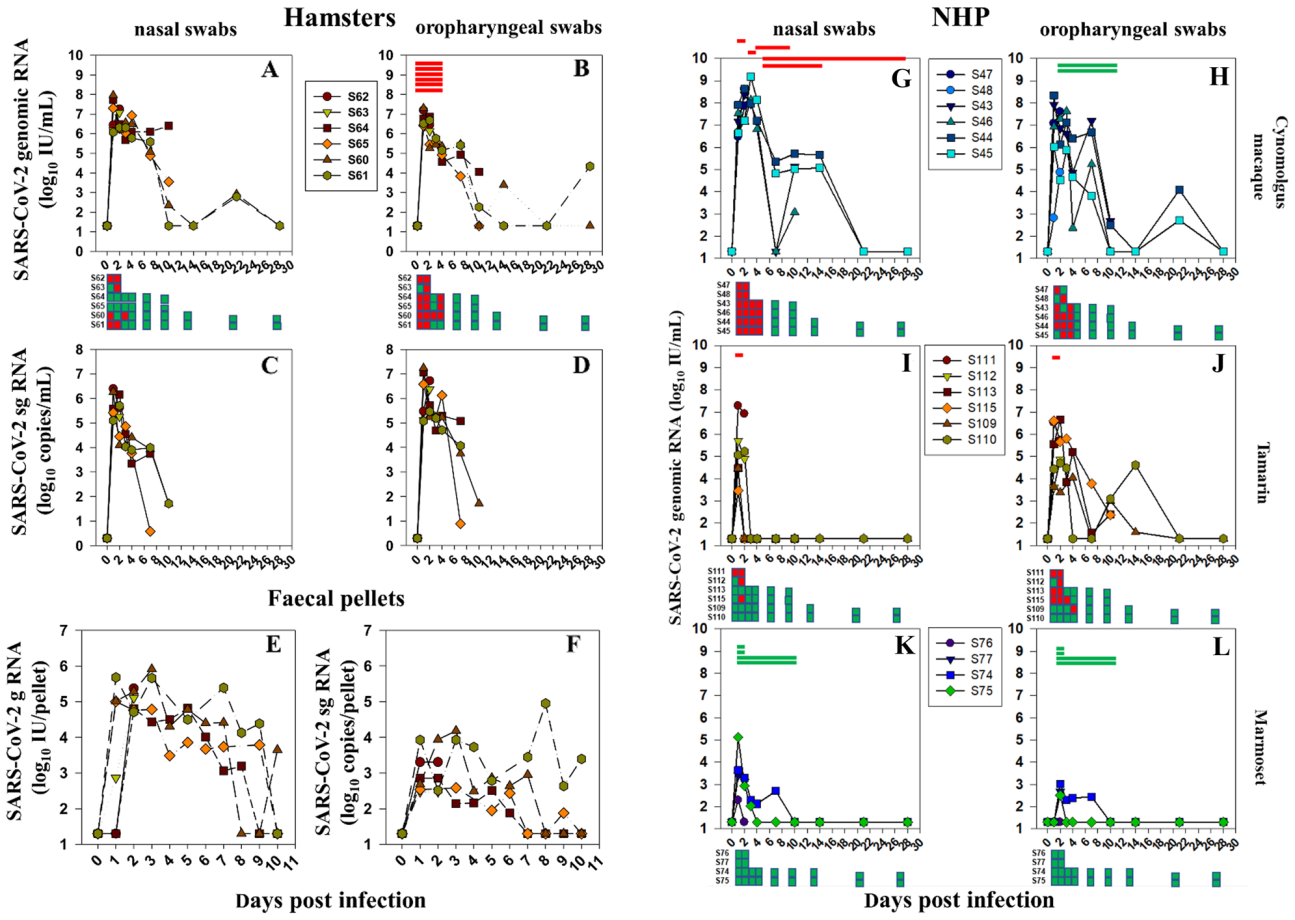
Infectivity and shedding profiles of SARS-CoV-2/Vic-01 in four independent species. (**A**, **B**) Comparative shedding outcomes in nasal and oral/mouth swabs in female hamsters followed over the time course. SARS-CoV-2 genomic RNA levels expressed as Log_10_ International Units (IU)/mL calibrated against the WHO International Standard for SARS-CoV-2 RNA (NIBSC# 20/146/Eng02) amplified with E gene primers in nasal and oral swabs monitored over the 28 day time-course. Qualitative microculture data using VeroE6/TMPRSS2 indicator cells shown in squares as green (no virus) and red (positive virus culture) at the times indicated. Red bars indicate positive antigen lateral flow device result where sampled. (**C**, **D**) Subgenomic (sg) RNA levels expressed as copies/mL in female hamsters over the acute period. (**E**, **F**) Genomic (g) and subgenomic RNA levels in faecal pellets recovered daily over the first 10 days in female hamsters. Comparable nasal and oral swab data are shown for the 3 NHP species: cynomolgus macaques (**G**, **H**), tamarins (**I**, **J**) and marmosets (**K**, **L**). Culture data are similarly indicated by red or green squares and lateral flow device result by red or green bars where sampled.

In vivo infectivity and pathogenesis of the SARS-CoV-2/Vic-01 P4 stock was compared across all four species, including male and female hamsters, monitored for viral shedding in nasal and oropharygeal swabs by genomic and sub-genomic RNA (gRNA, sgRNA), lateral flow antigen (nucleoprotein, NP) detection and infectious virus microculture (Figs. 2, S5, S6). Additional faecal shedding data was collected for female hamsters and cynomolgus macaques (Figs. 2, S7).

High levels of SARS-CoV-2 gRNA and sgRNA (range log_10_ 6–8 IU/mL gRNA, copies/mL sgRNA) detected in oropharyngeal or nasal swabs in female hamsters 2dpi had predominantly cleared 10dpi, (gRNA log_10_ 1–3 range), which remained undetectable with low, transient blips 21–28 dpi (S60, S61). The wide kinetic range of gRNA and sgRNA variation in intensively monitored naso- and oro-pharyngeal swabs in female hamsters was highly correlated during acute infection (Fig. 1E; correlation coefficient *r*^2^ = 0.98, *n* = 51; Fig. S5A,B). Antigen (NP) was consistently detected 1–4 dpi in oropharyngeal swabs (Fig. 2B). Upper respiratory tract (URT) shedding patterns were broadly similar between genders at both sample sites. However loads peaked 2 dpi at significantly higher levels in male nasal swabs compared with female (mean log_10_ 7.16 vs 6.44 gRNA IU/mL, respectively (Mann–Whitney rank sum test, p = 0.013), though only weakly significant 4dpi (one-tailed t-test, p = 0.114). In oral swabs, mean gRNA levels 2 dpi were not statistically different between females and males (mean log_10_ 6.82 and 6.69 IU/mL respectively), though weakly statistically significant 4dpi (one-tailed t-test, p = 0.103). Infectious virus was only recovered between 1 and 4 dpi, oral swabs more frequently positive in both sexes; 7/20 (nasal) and 15/20 (oral) swabs in females compared with 16/20 (nasal) and 17/20 (oral) swabs in males (Figs. 2C,D, S6). Post-acute (10dpi) viral shedding was low/undetectable in female hamsters, sporadic re-bounds at later time-points (S60, S61, d21 oral; S60, d14; S61, d28, nasal). Swab RNA dynamics compare with faecal pellets with sustained gRNA (range log_10_ 3–6 IU/pellet), lower but quantifiable sgRNA (range log_10_ 2–5 copies/pellet) over the first 10 days (Fig. 2E,F). Infectious virus was never recovered onto VeroE6/TMPRSS2 cells from faecal pellets though hamster S61 displayed an altered sgRNA profile and more persisting signal beyond 7 dpi.

Cynomolgus macaques exhibited the highest gRNA levels (range log_10_ 8–9 IU/mL) 2 dpi, in oral and nasal swabs (Fig. 2G,H), > tenfold higher in nasal swabs compared with contemporaneous oral swabs (log_10_ 8.23 vs 7.05 IU/mL), attaining statistical significance (Mann–Whitney test, p = 0.026). This differential relationship maintained 4 dpi (mean log_10_ 7.63 vs 5.82 IU/mL gRNA) co-incided with infectious virus recovery frequency 1–4 dpi. Differences in genomic RNA levels 10 dpi were log_10_ 5.28 and 2.32 IU/mL gRNA in nasal and oral swabs respectively, retaining statistical significance (p = 0.029, both). Macaques S44 and S45 both retained strong gRNA positivity 14 dpi (~ log_10_ 5–6 IU/mL), clearing 21 dpi with a small rebound in oral swabs 21 dpi. Antigen (NP) was frequently detected in nasal swabs, S44 remaining detectably antigen positive 28 dpi (Fig. 2G). Virus isolation was frequent 1-4 dpi from both nasal and oral swabs (20/20 and 16/20 respectively). As in hamsters, sgRNA levels were consistently ~ 1–2 logs lower than gRNA 2–4 dpi (Fig. S5C). Low, transient gRNA was detectable in rectal swabs in cynomolgus macaques (S45, S46, S48) but infectious virus was not recovered onto VeroE6/TMPRSS2 cells (Fig. S7).

Comparison of New World NHP species revealed differential susceptibility between marmosets and tamarins, but both lower than cynomolgus macaques. Notably, tamarins exhibited multiple markers of productive upper airway infection (Fig. 2I,J), peaking log_10_ 5–7 IU/mL gRNA in oral and nasal swabs with some persisting signals 14 dpi in oral swabs. Infectious virus was recovered from all tamarins from at least one time-point (1–3 dpi), more frequently from oral (9/20) than nasal swabs (4/20) that could not be attributed to in-put virus; NP antigen was also detected in tamarin S111 2 dpi at peak gRNA; sgRNA signals were detected in tamarins but at low levels. Marmosets had significantly lower peak gRNA 2 dpi, undetectable 7 dpi (Fig. 2K,L); neither antigen nor infectious virus were detected at any point in marmosets. Despite overall lower infectivity levels in marmosets, both S75 and S74 signalled for gRNA significantly above threshold, S75 peaking ~ log_10_ 5 IU/mL 2 dpi. Overall, applying multiple markers of decreasing virus infectivity, this Vic-01 passage 4 stock across the four species could be represented as:

$${\text{Cynomolgus }}\;{\text{macaques}} > {\text{hamsters}} > {\text{tamarins}} > {\text{marmosets}}$$

### Tissue biodistribution and timing of SARS-CoV-2 Vic-01 genome detection

**Figure 3:**
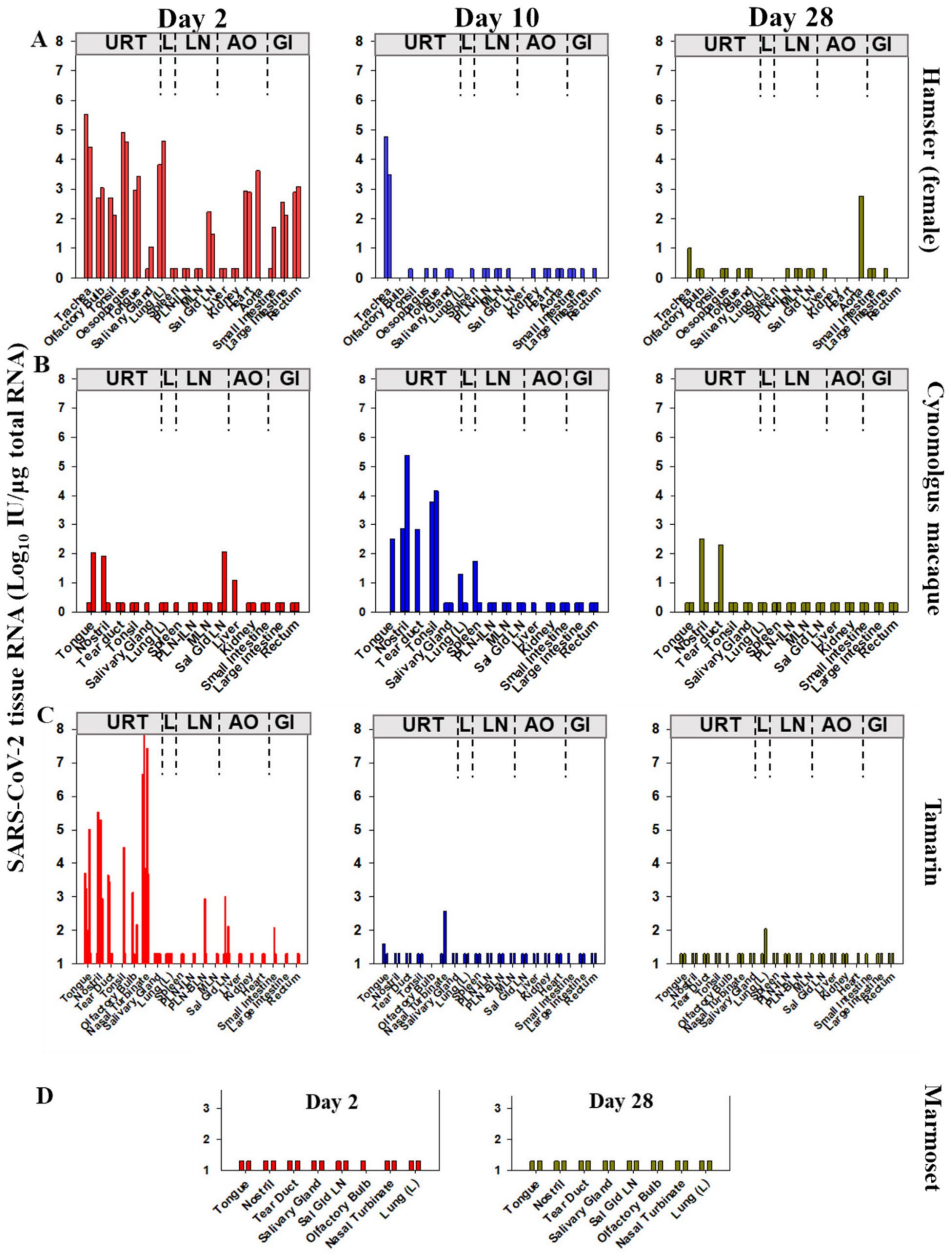
Anatomical biodistribution of SARS-CoV-2 genomic RNA. Multiple tissue extracts analysed for SARS-CoV-2 RNA expressed as Log_10_ International Units (IU)/µg total RNA. (**A**) female hamsters compared across the 2, 10 and 28 dpi time-course in multiple tissues from upper respiratory tract (URT), lung (L), lymphatic organs (LN), major abdominal organs (AO) and the gastrointestinal tract (GI); (**B**) cynomolgus macaque and (**C**) tamarins with a comparable set of tissues as indicated. (**D**) marmosets assayed at days 2 and 28 for a range of upper airway tissues and lung. Bars indicate tissues analysed which varied slightly across species; vertical bars up to the limit of detection of log_10_ 20 IU/µg total RNA indicate where a tissue was analysed but a negative result returned.

Comparison of viral genome biodistribution across multiple anatomical sites for each species using an assay expressing genomic RNA levels as International Units (IU)/µg total RNA (Fig. 3), indicated differences in timing and distribution between the upper and lower respiratory tracts and other sites across the time-course. In hamsters, all URT tissues sampled 2dpi were positive or strongly gRNA positive, as was lung tissue, salivary gland LN, heart, aorta, small and large intestine and rectum (Fig. 3A) reflecting the high virus shedding from multiple sites. Trachea, oesophagus and lung tissue all signalled > log_10_ 4 IU SARS-CoV-2 gRNA/µg total RNA, whereas the lymphoid system (spleen, mesenteric LN, peripheral LN) was genome negative. Heart and aorta tissue (S62) also signalled strongly, reflecting early widespread distribution and dissemination of SARS-CoV-2 in respiratory and cardiovascular systems but also the gastrointestinal tract (small and large intestines, rectum). At 10 dpi hamsters retained detectable gRNA only within the trachea (log_10_ 3–5 IU/µg RNA). By contrast, in cynomolgus macaques, tongue, nostril and salivary gland LN were sporadically genome positive 2 dpi compared to 10 dpi where gRNA signal increased in multiple URT tissues (e.g. nostril, tongue, tonsil). In both species 28 dpi, signals were only detected sporadically, nostril and tear duct positive in cynomolgus macaques and trachea and aorta in hamsters. Overall, gRNA persistence in cynomolgus macaques tissues reflected virus detection in swabs.

In New World NHPs, tamarins exhibited high levels of acute SARS-CoV-2 gRNA in nasal turbinates, nostril and tongue 2 dpi with lower levels in small intestine and salivary gland LN (Fig. 3C); only tamarin S110 signalled gRNA positive in lung 28 dpi. In contrast to tamarins, marmosets remained gRNA negative in all upper airway and lung tissues analysed 2 and 28 dpi (Fig. 3D). These data indicate early genome distribution and levels in tamarins to be at least comparable to hamsters, exhibiting high acute phase viral dynamics.

### Expression of nucleoprotein and spike antigens and RNAscope in hamster lung tissue

**Figure 4:**
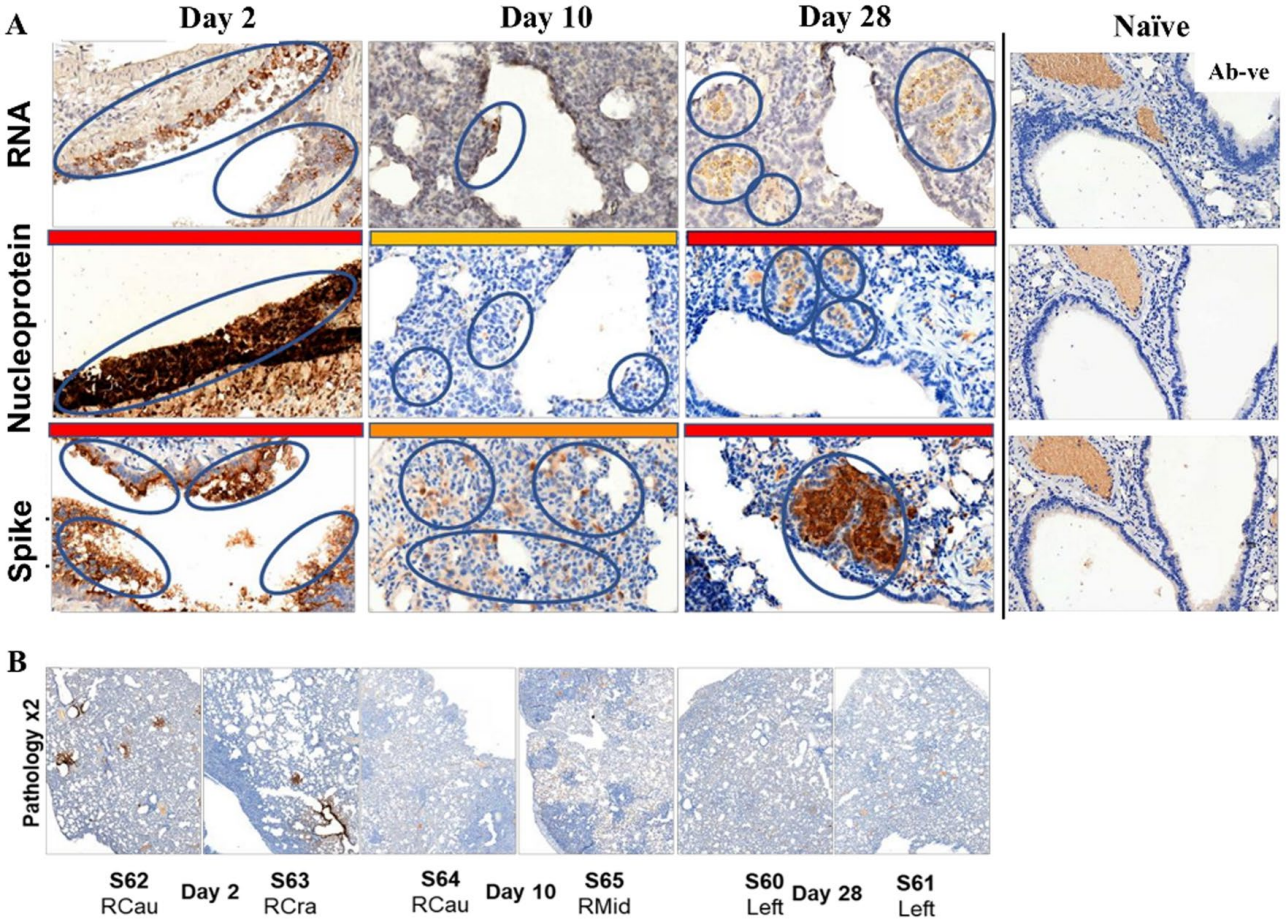
Detection and localisation of SARS-CoV-2 and sub-gross histopathological lesions in lung tissue. (**A**) RNAscope, nucleoprotein and spike protein detection in hamster lung sections across the 2, 10 and 28 day time-course. (**B**) sub-gross pathologic analysis of lung sections at each time point indicated. Brown labelling indicates nucleoprotein in lesions taken as a focus of infection of SARS-CoV-2. Day 2, RCau (right caudal), RCra (right cranial); day 10 RCau (right caudal), RMid (right middle). Labelling intensity indicated by red bars (high), orange bars (intermediate) and yellow bars (low) for IHC panels.

Rapid and extensive virus dissemination in hamsters was further characterized in lung tissue for relative abundance of viral NP and S antigens and viral nucleic acid (Fig. 4, females; Fig. S8, males). In females 2 dpi, high levels of viral S and NP antigen labelling by IHC and concordant detection of nucleic acid by RNAscope was located mainly within bronchiolar epithelial cells. At 10 dpi, all three markers were reduced compared with 2 dpi though more marked for anti-NP labelling, anti-S levels remaining within areas of inflammation. Notably, by 28 dpi, all three markers were at high levels, co-localising with areas of lung inflammation detectable by H&E staining. Histopathological changes across lung sections at each time-point with lesions corresponded to areas of brown labelling for nucleoprotein. It was not possible to interpret anti-S and anti-NP labelling within lungs of cynomolgus macaque, tamarin and marmoset due to antibody cross reactivity with antigens present in bronchiolar epithelial cells and vessel walls of lungs from naïve animals. Male hamsters exhibited concomitantly high levels of NP and S labelling with associated the pathological changes and more adverse clinical outcomes (Fig. S8).

### Early pathological changes following SARS-CoV-2/Vic-01 infection persist in hamsters

**Figure 5:**
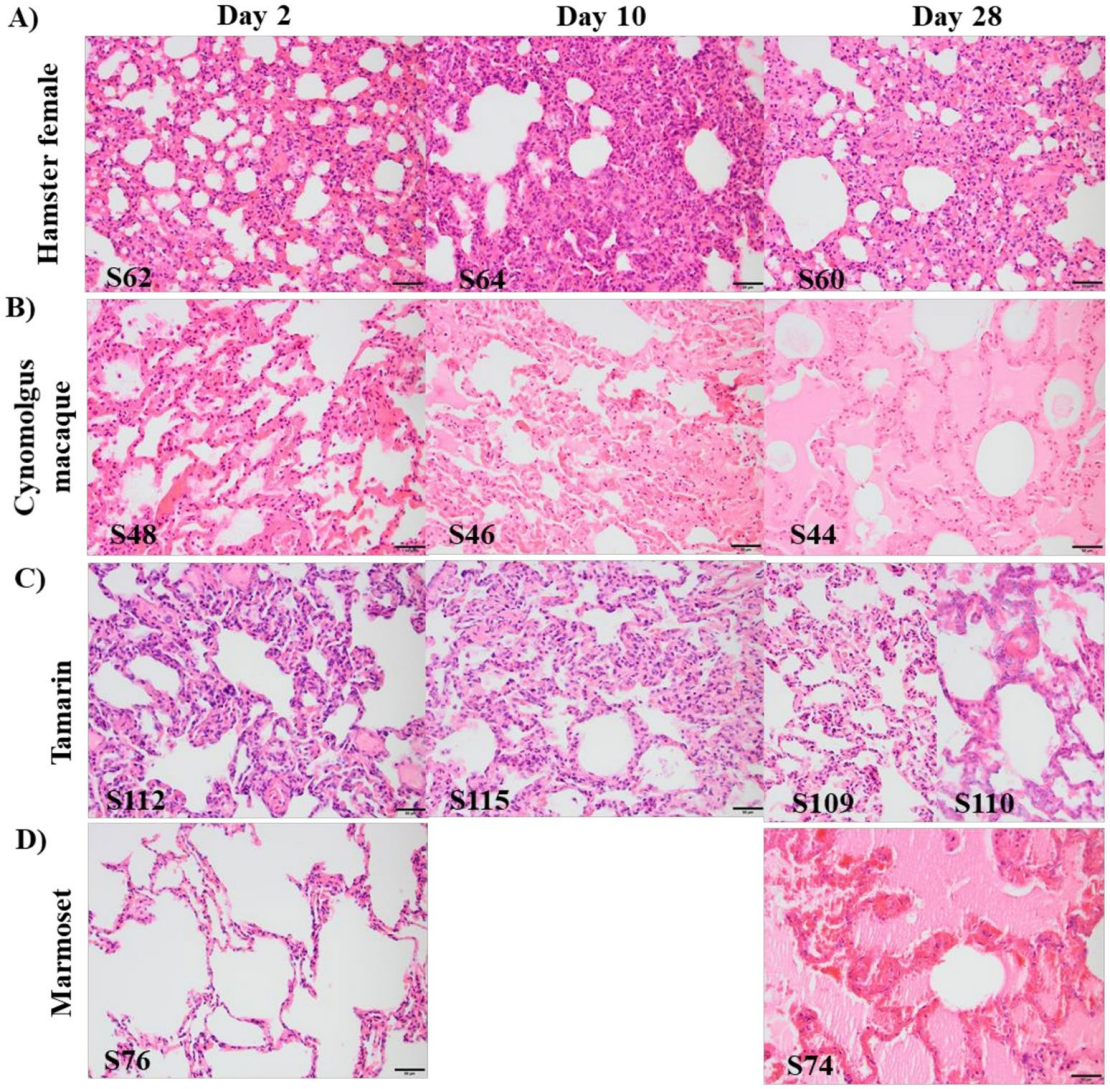
Comparative histopathological outcomes in lung section across the time-course of four species. Haematoxylin and eosin (H&E) staining of representative sections of lung tissue for: (**A**) female hamster lungs showing multifocally extensive alveolar wall congestion and mild to moderate protein-rich alveolar oedema. Foci of interstitial pneumonia with type II pneumocyte hyperplasia, and infiltrates of mixed inflammatory cells, including neutrophils, and single cell necrosis. (**B**) cynomolgus macaque; (S44, d28) diffuse, marked flooding of alveolar spaces with protein-rich oedema. (**C**) tamarin lung (e.g. S110, d28) typified by multifocal alveolar wall degeneration and necrosis with multifocal organising fibrin exudation. Alveolar wall necrosis, abundant fibrin exudation and hyaline membrane formation. (**D**) marmoset, showing multifocally extensive alveolar wall necrosis, intra-alveolar fibrin exudation and haemorrhage at day 28 (S74). Alveolar wall necrosis, abundant fibrin exudation and early hyaline membrane formation were present. Bar represents 50 µM.

**Figure 6:**
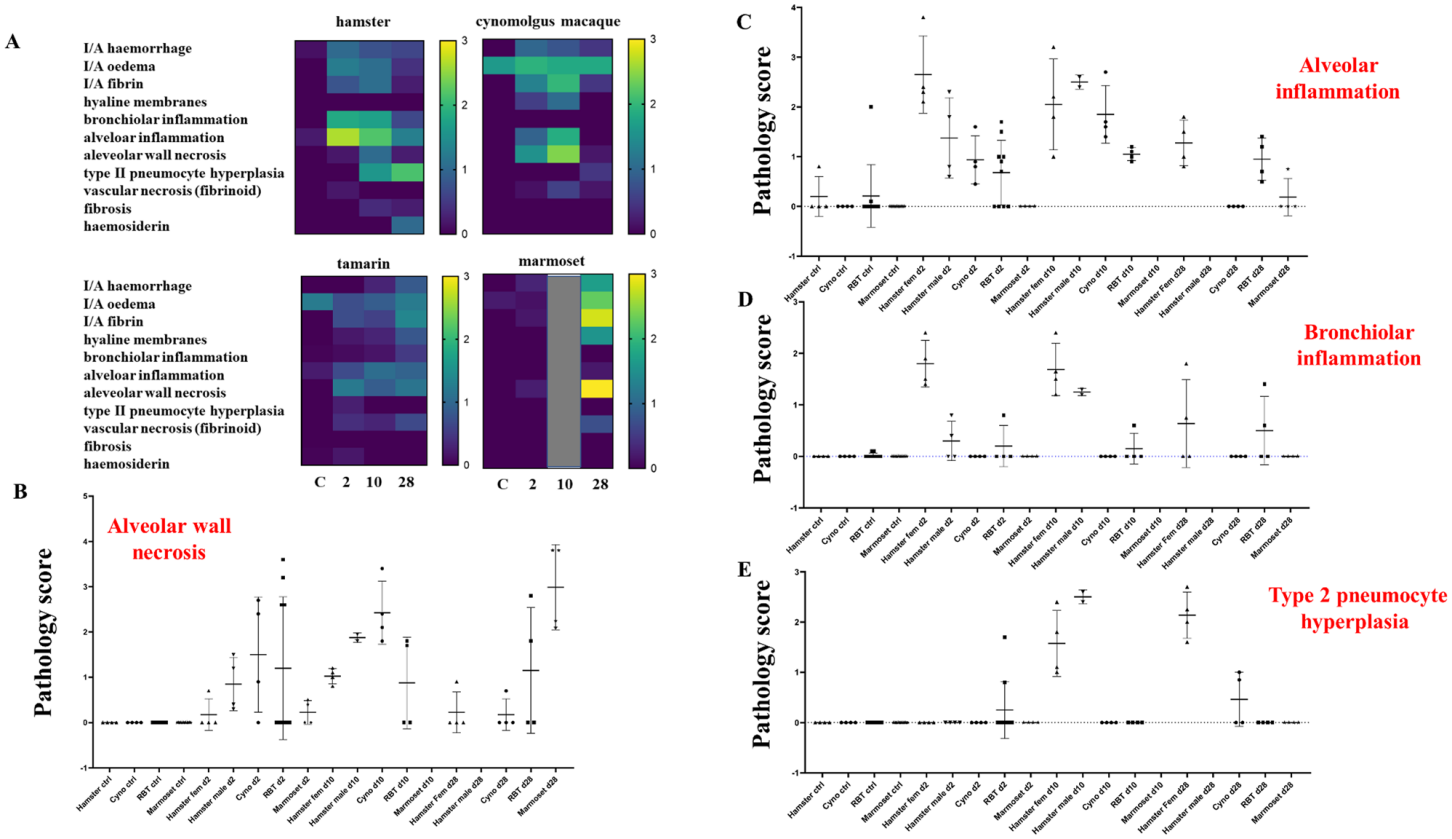
Comparative pathology scores in four species across the time-course. (**A**) histopathology outcome heat map scores in each species: 0 = minimal, 1 = mild, 2 = moderate, > 3 = marked. (**B**–**E**) percentage adjusted scores of overall pathology for each of alveolar wall necrosis, alveolar inflammation, bronchiolar inflammation and type 2 pneumocyte hyperplasia across the time-course for each of the four species. *Cyno* cynomolgus macaque, *RBT* red-bellied tamarin. Heat map graphics generated using GraphPad Prism version 9.3 software.

Lung histopathology following SARS-CoV-2 infection was determined for female and male hamsters by haematoxylin and eosin-staining (Figs. 5A, S9); key pathology markers scored blind by two board-certified veterinary pathologists are presented in heat-map format (Figs. 6A, S9). Percentage adjusted data for four key histopathologic indicators (alveolar wall necrosis, alveolar and bronchiolar inflammation and type 2 pneumocyte hyperplasia) were compared across species taking into account lesion distribution across the tissue section. Significant evidence of altered vascular permeability/damage and blood coagulopathy 2dpi was linked with haemorrhage, inflammatory cell infiltrates and mild to moderate fibrin exudation with acute histopathological lesions and bronchiolar and alveolar inflammation. Some resolution was evident 10 dpi, though moderate/marked bronchiolar and alveolar inflammation, fibrin deposits and haemorrhage remained accompanied by type II pneumocyte hyperplasia. Hamster S61, with detectable viral RNA 28 dpi and strong IHC immunostaining with anti-S and anti-NP antibodies exhibited multifocal evidence of type II pneumocyte hyperplasia, mild fibrosis, low levels of alveolar and bronchiolar inflammation and most other descriptors of lung pathology; formation of hyaline membranes was not observed at any time-point.

In male hamsters, more extensive histopathological changes were observed humanely sacrificed 10 and 14 dpi (Fig. S9). Early, high levels of virus shedding and IHC lung staining for viral antigens was associated with more extensive and adverse clinical scoring especially alveolar wall inflammation and necrosis, type-2 pneumocyte hyperplasia, excessive fibrin exudation and haemorrhage.

### SARS-CoV-2/Vic-01 lung pathology outcomes in Old and New World NHP species

Comparative lung pathology in Old World cynomolgus macaques and New World tamarins and marmosets are summarised in Figs. 5B–D and 6 indicating early changes in lung pathology scores across species. In cynomolgus macaques, 2 dpi there was evidence of haemorrhage, fibrin deposits and alveolar inflammation; by 10 dpi overall pathology scores had increased with more significant alveolar inflammation and alveolar wall necrosis, though had mostly resolved 28 dpi. Lung histopathological changes determined for all three time-points in tamarins were generally mild; alveolar wall necrosis and inflammation mild to moderate, contrasting with marmosets which displayed more marked lung pathology 28 dpi. Within each NHP species more extensive pathology appeared related to overall levels of virus replication, whether due to a more aggressive acute infection profile or failure to fully clear virus at later times. Macaque S44, tamarin S110 and marmoset S75 all demonstrate this association, exhibiting significant SARS-CoV-2-related pathology 28 dpi. All 3 NHP species identified with early and sustained lung pathological consequences following intranasal-only virus challenge.

### Morphological changes in cellular organelles in lung tissue across multiple species

**Figure 7:**
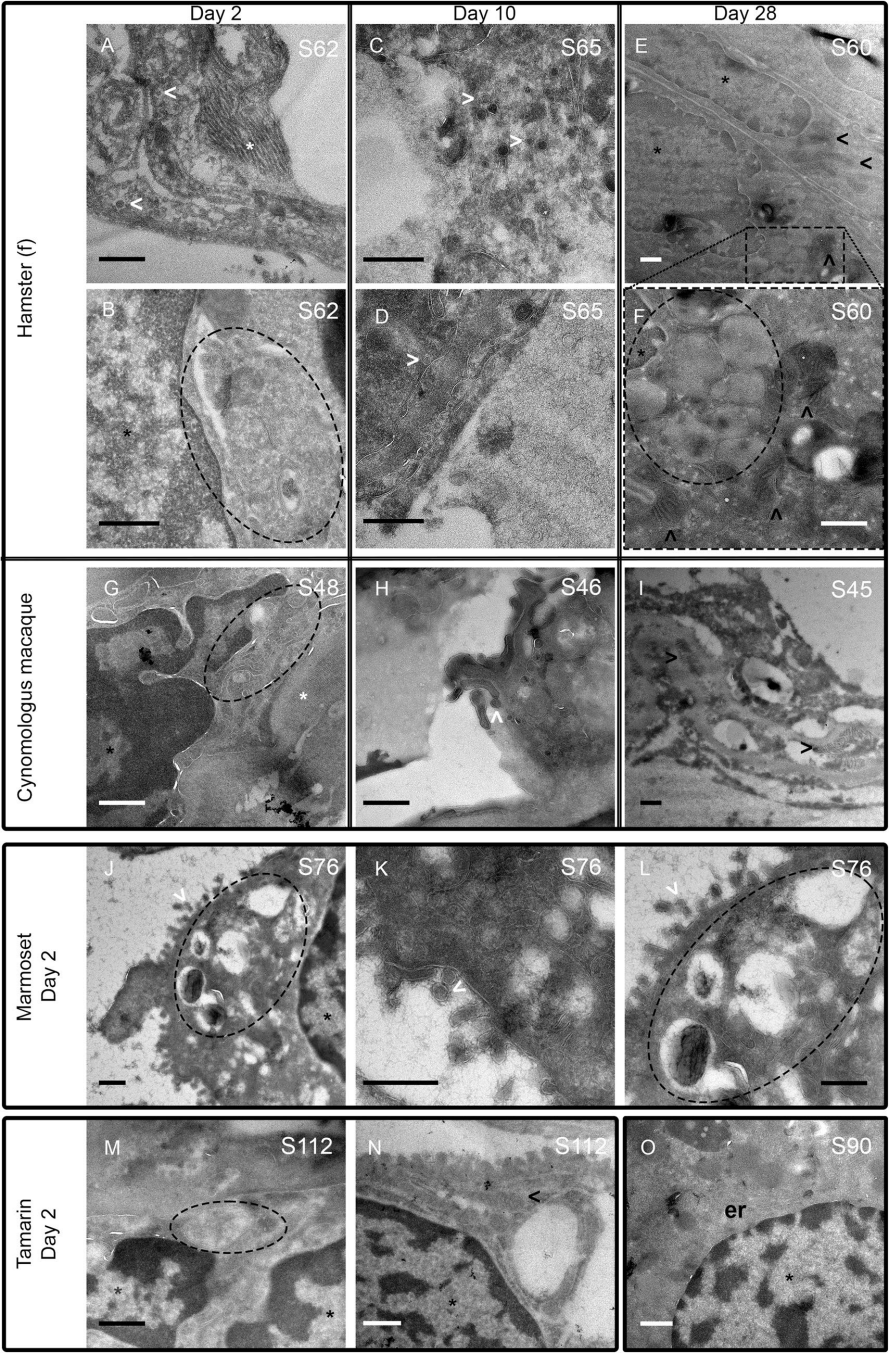
Tokuyasu cryo sections of lung tissue. At day 2 in hamster lungs (S62), virions (white open arrowheads) were visualised (**A**) and associated with perinuclear replication organelles (dotted ellipses) (**B**). At 10 dpi (S65) SARS-CoV-2 virion-like particles were further identified in lung tissue (**C**) with evidence of possible budding (**D**). Differences in morphology between infected cells and cells without current infection are apparent in hamster S60 extending out to 28 dpi (**E**) with replication organelles visible in infected cells (bottom cell). (**F**) (S60) is a higher magnification of E showing the replication organelle and mitochondria (black open arrowheads) with evidence of disrupted cristae indicating more generalised disrupted cell ultrastructure. (**G**–**I**) cynomolgus macaques S48, S46, S45 at days 2, 10 and 28 respectively. Virions shown (day10) with evidence of disrupted mitochondria 28dpi (panel **I**). (**J**–**L**) membrane-associated virions identified in marmoset S76 2 dpi. (**M**, **N**), tamarin lung S112 at day 2 with evidence of virions associated with a replication organelle. (**O**) uninfected hamster lung (S90) displays long sections of endoplasmic reticulum (er) in the perinuclear region. Magnifications range from 6000 × to 25,000 × with all scale bars corresponding to 500 nm. Annotations correspond to white open arrowheads, virions; black open arrowheads, mitochondria; and dotted ellipses, replication organelle. White asterisk, back asterisk and er denote collagen fibrils, nucleus and endoplasmic reticulum respectively.

Ultrastructural analysis of lung tissue was undertaken by electron microscopy to assess the integrity, morphological changes and localisation of key cellular organelles (Figs. 7, S10). In hamsters (Fig. 7A–F), virions were identifiable in lung sections, associated with perinuclear replication organelles 10dpi when viral detection in the URT had largely resolved (Fig. 7A,B). Differences in morphology between infected and uninfected cells was observed at all timepoints associated with replication organelles. Evidence of mitochondrial disruption, with altered cristae presentation was further associated with generalised disrupted cell ultrastructure at later times (day 28). Images for macaques (Fig. 7G–I) show virions identified in the replication organelle of cynomolgus macaque (e.g. S46). By 28 dpi more extensive evidence of mitochondrial damage was seen in the proximity of replication organelles. Direct identification of virus in lung tissue, longer term effects of virus infection on key cellular organelles in both hamsters and cynomolgus macaque appeared a common feature, with evidence of disrupted cellular organelles within infected and uninfected regions (Fig. 7I). In New World species, virus associated with nuclear membranes was observed in marmoset lung 2 dpi (S76) (Fig. 7J–L), suggestive of some transient virus replication in lung tissue. In both tamarins and marmosets, virus-like structures associated with replication organelles in lung tissue were observed by EM, providing direct evidence of rapid virus dissemination to this key site of SARS-CoV-2 pathology following intranasal virus exposure.

### Variable anti-SARS-CoV-2 responses across species

**Figure 8:**
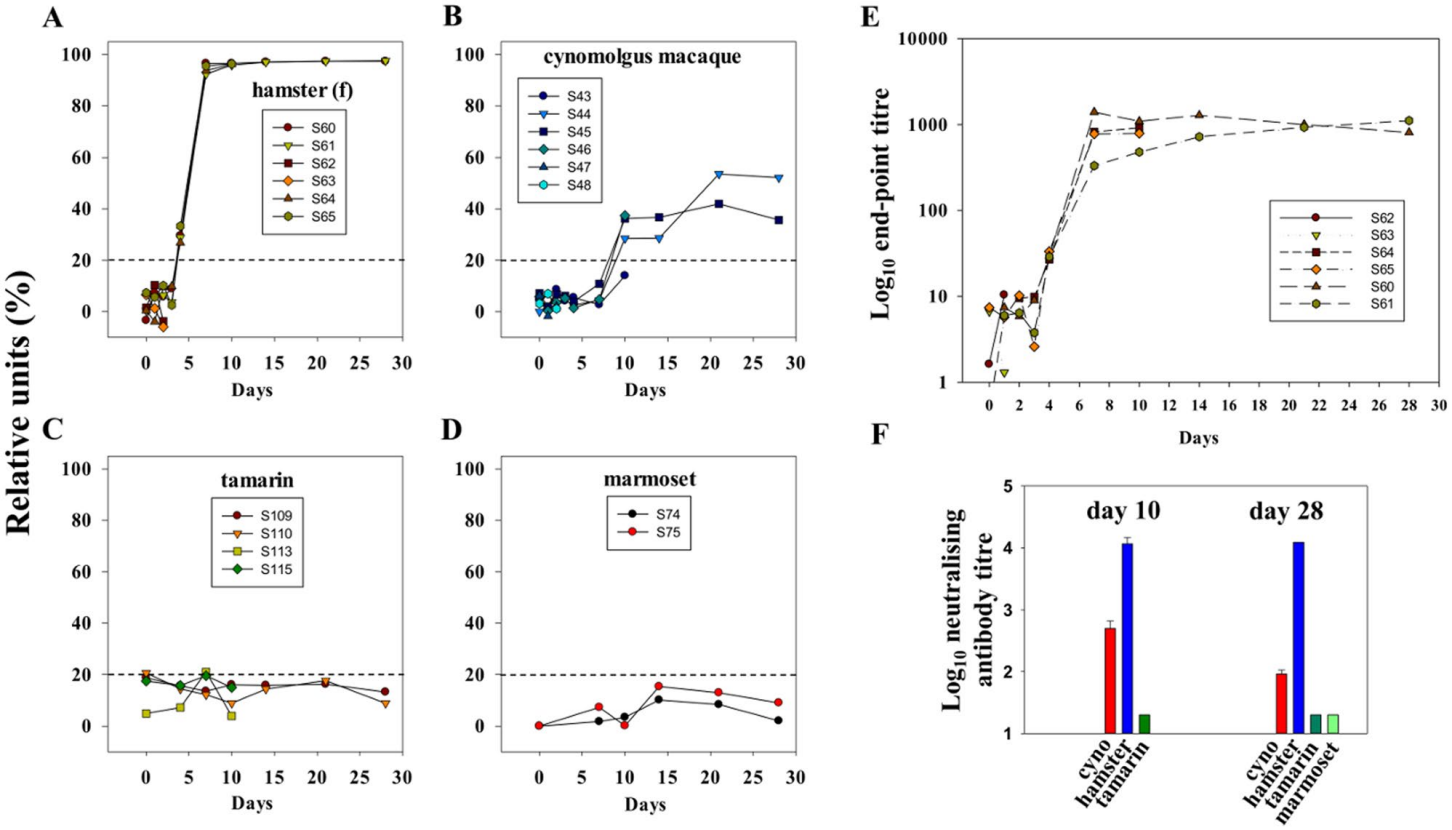
Time-course of anti-SARS-CoV-2 responses across species. Anti-RBD antibodies detected by the Genscript surrogate competition ACE-2 neutralisation (C-ACE-2) assay expressed as percentage inhibition (relative units) for (**A**) female hamsters, (**B**) cynomolgus macaque, (**C**) tamarin, (**D**) marmoset 0-28dpi. Dotted lines indicate assay cut-off of specific reactivity. (**E**) Log_10_ end-point titers measured in hamster sera by C-ACE-2 assay, (**F**) titres by microneutralisation assay (MNA) 10 and 28 days post-infection for hamsters (blue), cynomolgus macaques (red), tamarins (dark green) and for marmosets at day 28 (light green).

Antibody responses across species were assessed using a species-independent competitive ACE-2 (C-ACE) assay to compare anti-RBD antibody responses and neutralising antibodies by live Vic-01/virus microneutralization assay (Fig. 8). By C-ACE-2 inhibition assay, hamsters signalled strongly (100% inhibition 7dpi) at early time-points (Fig. 8A) indicative of rapid seroconversion. All 6 cynomolgus macaques also seroconverted, responses developing later 10–28 dpi (Fig. 8B); the highest percent inhibition by C-ACE-2 assay in macaque S44 correlated with persisting antigenaemia and gRNA detection. No sera from New World marmosets or tamarins signalled at any time-point above cut-off in the C-ACE-2 assay, (Fig. 8C,D). Female hamsters analysed by end-point titration in the C-ACE-2 assay indicated titres rising rapidly to plateau at log_10_ 3 at 7 dpi (Fig. 8E). Sera collected 10 and 28 dpi tested by microneutralisation against Vic-01 confirmed data from the C-ACE-2 assay (Fig. 8F). Overall, very high titres (~ log_10_ 4) were observed in hamsters, intermediate titres (log_10_ 2–2.6) in cynomolgus macaques and no neutralising activity in either New World species.

## Discussion

Clinical presentation of human SARS-CoV-2/Covid-19 infection and disease varies considerably. Accounting for this diversity in animal models represents a key part in experimental design of pre-clinical studies of SARS-CoV-2, where different outcomes across species may provide conflicting interpretations. Using standardized methodologies, a well-characterised virus stock and common study plan we performed robust head-to-head comparisons of three NHPs (cynomolgus macaque, red-bellied tamarin, marmoset) and one small animal (Syrian hamster) following non-invasive, I.N.-alone administration of the SARS-CoV-2/Victoria strain. Minimising inter-species anatomical differences and using the same inoculum dose and route of virus administration in all four species, diverse infectivity, virological and pathological outcomes were identified to reflect much of the range observed in humans, with pathophysiological features resembling human Covid-19 disease in hamsters to sub-clinical infection in New World monkey species. Notably, New World tamarins were identified as being susceptible to SARS-CoV-2. Despite species-specific susceptibility to infection, differing replication dynamics and virus sequestration when introduced intranasally, lung pathological consequences that extended to ultrastructural changes which impacted on cellular organelle integrity were observed across all species. In these predominantly asymptomatic models of SARS-CoV-2 infection, such alterations to lung organelle ultrastructure underscore wider pathological consequences after clearance of viral shedding.

While all Old World NHPs appear susceptible to SARS-CoV-2 infection, variable disease outcomes seem dependent on delivery route whether virus has been administered via combined I.N./I.T., sometimes in conjunction with other mucosal sites (e.g. ocular, oral) or an aerosol route^27–31^. Omittance of I.T./multi-mucosal virus routes allows some alignment of outcomes following aerosol inhalation of virus, with I.N-only administration identifying differences in timing and distribution of virus dissemination between NHP species and hamsters. In these Indonesian cynomolgus macaques, IN.-only virus yielded high levels of acute upper airway virus replication (peak ~ log_10_ 9 gRNA IU/mL), greater than Syrian hamsters when compared using standardised genomic RNA assays for swabs and tissues. Indonesian cynomolgus macaques exhibit more outbred immunogenetic profiles than Mauritian-derived animals^32^, which perhaps accounts for divergent patterns of post-acute virus control and may better reflect differences in individual host susceptibility^33^. Comparative baseline data for Vic-01-infected hamsters confirmed more severe clinical, virological and histopathological manifestations, typified by high acute virus in upper and lower respiratory tracts 2 dpi, rapid, widespread genome dissemination impacting respiratory, cardiovascular and gastrointestinal systems. While this acute response profile may not typically reflect human SARS-CoV-2 infection kinetics, differences in anatomical distribution of viral genome and virus kinetics between different species was evident using the same virus dose; acute disseminated Vic-01 biodistribution in tissues cleared 10 dpi in hamsters compared with more persisting gRNA in upper airway tissues of cynomolgus macaques.

Hence, many factors confer susceptibility and virulence of SARS-CoV-2 across diverse mammalian species. ACE-2 sequence homology across RBD/S remains an important consideration, though an absolute relationship does not exist. Pigs (*Sus scrofa*) should support SARS-CoV-2 replication based on ACE-2 predictions, but independent studies demonstrate determined host resistance by multiple infection routes and challenge regimes^34^; other species, (e.g. cows, dogs, cats) exhibit variable susceptibilities to experimental infection^35,36^. Fewer in vivo studies of *Platyrrhine* family members exist^37^, though New World NHPs appear more refractory to SARS-CoV-2 compared with Old World species^14,37^. In our study, high shedding and tissue dissemination profiles in hamsters and macaques contrasted with New World marmosets receiving the same virus dose via the same route. By contrast, tamarins supported high levels of virus in upper respiratory tract swabs and tissues, transient recovery of infectious virus by co-culture on VeroE6/TMPRSS2 cells portraying an intermediate picture between macaques and marmosets associated with identifiable lung pathology. Our marmoset findings broadly concur with studies delivering relatively high dose/volumes of virus via the I.T. route^14^, indicative of an intrinsically more refractory species to SARS-CoV-2 infection. Interestingly, we did detect virions in marmoset lung tissue 2 dpi by EM with evidence of long-term lung pathology 28 dpi suggestive of some initial up-take of virus in this species.

Tamarins therefore present with a different outcome to represent a hitherto unrecognized New World species susceptible to SARS-CoV-2, supporting virus replication in the upper airway. Multiple URT tissues were strongly gRNA positive, with other biomarkers (e.g. infectious virus recovery, antigen) indicative of some on-going, acute infection. Intriguingly, ACE-2/RBD sequence homology is similar between these two closely related species, tamarin ACE-2 possessing at least one amino acid (T27A) mutation under positive selection in bats (*Chiroptera* spp.)^6^. Productive upper airway infection of red-bellied tamarins, indigenous to Amazonian South America highlights potential exposure and transmission risk of a susceptible species with proximity to human populations that experienced high levels of SARS-CoV-2 exposure^38,39^. Transfer of virus into otherwise naïve animal reservoirs remains a concern, since North American white-tailed deer susceptible to SARS-CoV-2 have a seroprevalence of ~ 40%^40^, deer mice exhibit evidence of sustained populational virus exposure^41,42^ and mustelids (e.g. mink) can be infected with SARS-CoV-2 leading to clinical disease^43,44^. Potential for reverse zoonoses of SARS-CoV-2 into susceptible species may therefore be more common than hitherto anticipated, re-introduction into human populations posing a potential risk if adaptive virus evolution has occurred.

The administration of a single, common dose to the four susceptible species highlights differential outcomes to SARS-CoV-2 infection characterized by variable virus clearance, biodistribution and pathology. The use of a common dose is essential for species comparison studies since different outcomes have been reported in hamsters relating to the infectious dose administered^45^. Here, four species exposed to the same viral dose, displayed pathology outcomes 28 dpi that reflect potential long-term effects even in apparently less susceptible hosts. Conventional lung pathology coupled with alterations in ultrastructural integrity identified by electron microscopy 10–28 dpi in post-acute/chronic phases indicate an impact on lung integrity extending beyond times when the majority of virus has cleared from most sites. Coronaviruses induce membrane re-arrangements in order to replicate efficiently and assemble^46–48^. However we showed that species supporting virus replication during the acute phase exhibited longer-term physiological changes to lung cellular organelles, including mitochondria, with evidence of virus associated with replication organelles and generalised ultrastructural disruption. Interestingly, virions in tamarin lung tissue 2 dpi by EM were less evident, possibly explaining some pathology score differences 28 dpi between marmosets and tamarins. The wider impact of the virus on mitochondrial integrity and other key cellular organelles, implies underlying mechanistic effects of physiological function after SARS-CoV-2 infection^49^ in otherwise asymptomatic infections. Full resolution of these morphological changes may be crucial to a return to normal physiological function.

A number of infection biomarkers were employed in this study to compare outcomes. Harmonisation of data was further facilitated by incorporation of International Standards and reference materials (e.g. genome and/or antibody) to bridge between animal and human studies^50^. Genomic RNA read-outs expressed in IU/mL against the WHO IS for SARS-CoV-2 RNA allows for potential harmonisation of gRNA data. Standardised gRNA correlated with sgRNA at different stages of acute/post-acute (1–10 days) infection, the latter representing a possible surrogate biomarker for infectious virus^51^. The advantage of the WHO IS material is that unlike multiple sgRNA target sequences (e.g. N, E, Orfs), comparative data is more readily generated. Interestingly, ORF-7 sgRNA levels detectable in hamster faecal pellets over this period correlated with higher levels of sustained gRNA signals, yet infectious virus was never recovered reflecting wider observations of an apparent absence of infectious SARS-CoV-2 in faecal material despite widespread presence of SARS-CoV-2 RNA in wastewater^15,52^. Hence, different infectivity biomarkers at multiple sampling sites provide the fullest picture of active virus replication, in conjunction with detection of viral antigen expression in key target tissues; NP rather than S staining in lung tissue better reflected changes in antigenic expression over the time-course in hamsters.

New World hosts which fail to seroconvert may be a feature of this group, with variable antibody responses detected across all species studied. Natural host resistance to infection and dose response characteristics likely overlap in any given species as seroconversion to aerosol administered virus in cynomolgus macaques has been shown to be dose-dependent^28^ and a minority of humans fail to seroconvert during natural infection with abortive or subclinical infection linked to enhanced T-cell surveillance in the absence of classical infection markers^33^. Antibody responses against non-RBD, off-target epitopes and non-neutralising responses would clarify serological responses in New World species but clearly contrasts with rapid, robust seroconversion in hamsters to RBD/S 7–10 dpi; macaques displayed intermediate responses more typical of human antibody profiles.

Insights into the widest host range susceptibility to SARS-CoV-2 will further inform our understanding for potential novel animal reservoirs to be generated, the ecology and pathogenesis of novel SARS-CoV-2 variants further influenced as the virus evolves under different selection pressures. As novel variants continue to emerge, where evolutionary pressures impact on interactions with ACE-2 and TMPRSSS2, this work provides a baseline for on-going VOC characterization, particularly in the hamster model. As the longer-term impact and wider consequences of otherwise asymptomatic SARS-CoV-2 becomes better recognized, development of novel interventions aimed at minimizing these effects in non-hospitalised patients may be accelerated in these asymptomatic hosts, providing a robust scientific regulatory framework for evaluation of effective virus countermeasures.

Moreover, our data underline the need for on-going refinement of animal models for SARS-CoV-2 as a pre-requisite for pre-clinical testing evaluation of candidate prophylaxis and disease treatments, including vaccines, defining correlates of protection and immunity and to elucidate the longer term pathological effects of SARS-CoV-2.

## Materials and methods

### Virus stock preparation and deep sequencing

The Victoria-01/2020 isolate, originating from the Doherty Institute, Melbourne, Victoria, Australia was obtained via UKHSA/Porton, UK at passage #3, propagated once more at NIBSC at MOI 0.001 on Vero/hSLAM or VeroE6/TMPRSS2 cells to derive two independent stocks of Vic-01. All animals were challenged with a bulk passage #4 stock grown on Vero/hSLAM cells with a final titre of 2 × 10^6^ TCID_50_/mL. Next generation sequencing for amplicon-based recovery of SARS-CoV-2 RNA using Illumina technology, generated deep sequence to confirm the genetic identity as wild type virus and lack of attenuating mutations in the furin cleavage site and elsewhere.

Sequencing the VeroE6/TMPRSS2 expanded stock was made using 30 overlapping primer pairs^53^; for the Vero/hSLAM stock additional sequence for the terminal amplicons were extended using nCOV2019_1_LEFT and nCOV2019_98_RIGHT from ARCTIC nCOV-2019 V3 panel^54^ to pair SARS-COV-2_1_R and SARS-COV-2_30_F respectively to generate a near complete sequence. Sequencing libraries were constructed using the DNA Prep kit (formerly known as Nextera DNA Flex) and IDT for Illumina Nextera DNA unique dual indexes (both Illumina, San Diego, CA, USA), as per manufacturer’s instructions. Sample purification bead master mix was adjusted to 1:1 ratio to produce libraries with a larger insert size.

Libraries were subsequently pooled and sequenced on an Illumina MiSeq platform at 250 bp paired-end reads (Illumina). Initial de-multiplexing and FASTQ conversion was performed on-board by the MiSeq reporter software.

### Bioinformatics

Data generated from overlapping amplicon fragments were analysed using a bespoke bioinformatics pipeline incorporating Lofreq and iVar^55^ variant calling programmes. Illumina MiSeq generated sequence data from individual amplicons, trimmed and aligned against refseq_NC_045512_covid19_Wuhan, then sequence output merged. Variants were called using iVar and LoFreq separately, then collated. Sequence files are deposited as an SRA biosample submission under entries: SAMN18632196 SARS‐CoV‐2/human/AUS/VIC01/2020 https://www.ncbi.nlm.nih.gov/biosample/SAMN18632196 SRA and SAMN18632233 SARS‐CoV‐2/human/AUS/VIC01/2020 https://www.ncbi.nlm.nih.gov/biosample/SAMN18632233 SRA.

### Ethics statement

All animal procedures and protocols were conducted in strict accordance with UK Home Office guidelines, under a licence granted by the Secretary of State for the Home Office which approved the work described. Animal work at NIBSC is governed by the Animals (Scientific Procedures) Act 1986 that complies with the EC Directive 86/609; this study was performed under a licence (PPL2209804) granted only after review of all procedures by the NIBSC local Animal Welfare and Ethical Review Body and in accordance with ARRIVE guidelines. All experimental protocols and procedures were approved by the UK Home Office and NIBSC AWERB committee in light of best current practice for SARS-CoV-2/Covid-19 animal experimentation, guided by the most recent expertise shared through expert group meetings (e.g. WHO animal model expert group). This was particularly the case for experimental work involving hamsters for interpretation of clinical outcomes and animal welfare. For NHPs, regular modifications to the housing area have been made by husbandry staff including availability of novel structures (e.g. swings and perching stations) and foodstuffs in novel manners to encourage foraging for food, to further enrich the study environment. The environmental temperature (15–24 °C), was appropriate and rooms were subject to a 12 h day/night cycle of lighting. Animals were acclimatised to their environment and deemed to be healthy by the named veterinary surgeon prior to inclusion on the study. NHPs were sedated with ketamine prior to bleeding or virus inoculation by venepuncture. Frequent checks were made by staff and any unexpected change in behaviour by individuals on study followed up, including seeking of veterinary advice where necessary. All efforts were made to minimise animal suffering, including provision of a high standard of housing quarters, monitoring of animal health and well-being and absence of procedures not essential to the study.

### Challenge study design

A final challenge titre of 5 × 10^4^ TCID_50_ of the deep sequenced passage 4 SARS-CoV-2/Victoria-01 stock was administered atraumatically via the intranasal (I.N.) route to each of 12 Syrian golden hamsters (6 female, 6 male); 6 cynomolgus macaques (female) 4 common marmosets (2 male, 2 female) and 9 red-bellied tamarins (1 male, 8 females) according to the study plan summarized in Fig. 1A. 50ul final volume of the 2 × 10^6^ TCID_50_ stock was administered to each hamster. New World NHPs were challenged with the same dose/volume as hamsters, 50 µL administered volume to marmosets and tamarins, with 50 µL virus in 500 µL final volume administered to macaques taking into account the inter-species size difference. All challenge protocols and practices were regularly reviewed by veterinarian and animal husbandry staff at NIBSC.

### Clinical monitoring

During a one-week pre-challenge acclimatisation period, remote transponder chip implants (IPTT-300) were surgically delivered to hamsters by sub-cutaneous injection to provide a measure of body temperature. Baseline weight loss/gain measurements were determined for individual hamsters following a 7-day monitoring period of weights prior to challenge, to obviate individual weight variations. Weight loss at day of challenge was taken as a baseline reading. Virus was administered directly to the nostrils by pipette (50 µL total volume equally distributed between nostrils) representing 5 × 10^4^ TCID_50_ viral stock inoculum as the final administered dose. Hamsters were weighed twice daily (am, pm) and monitored for clinical signs. Clinical scores were recorded using a range of criteria such as behaviour, appearance (ruffled fur, piloerection), breathing response and ear position. Temperature fluctuations in NHPs were monitored by implanted devices that transmitted data to a receiver every 30 s, captured and an average temperature per hour calculated for each individual. All four species were humanely sacrificed in conjunction with a full cardiac bleed performed under terminal/surgical sedation (ketamine/xylazine) with an overdose of a pentobarbitol solution (Dolethal/Euthatal) via the cardiac route.

### Sampling and tissue recovery

Throughout the study period, intensive sampling by means of nasal and oropharyngeal sites were taken from each animal at days 0, 1, 2, 3, 4, 7, 10, 14, 21 and 28 post challenge. Swabs were taken into 1.2 mL Virus Transport Medium (VTM; Viral Transport Media: Hanks Buffered Salt Solution (Gibco), 2% FCS, 0.5 µg/mL Amphotericin B, 100U/mL penicillin/streptomycin). Rectal swabs from cynomolgus macaques and faecal pellets were collected sequentially from hamsters following virus challenge. At post-mortem, intact lungs and a broad tissue list were harvested from major anatomical sites of upper respiratory tract, lung tissue, the major lymphatic organs, abdominal organs and the gastrointestinal tract as detailed in Fig. 3. For PCR analyses both fresh frozen and RNA later (Qiagen) aliquots were preserved for PCR analyses according to manufacturer’s recommendations. Lung tissue was fixed for 72 h at room temperature in 10% formal saline (Sigma) and embedded in paraffin wax (VWR) in accordance with standard histological processes.

### Quantitative RT-qPCR analysis of swabs

Oro- and nasopharyngeal swabs were subjected to RT-qPCR analyses for presence of viral genome. The limit of detection for viral genomic RNA was determined to be log_10_ 50 IU/mL. Comparison of regression data for the WHO IS NIBSC 20/1456 and the Vic-01 standard series are shown in Fig. S11. Total nucleic acid was extracted from 200 µL using the MagnaPure24 (Roche) External Lysis Pathogen 200 protocol with a final elution into 50 µL. Quantitative measurements were made using a working panel of the VIC-01 isolate (NIBSC# 100987) derived by ten-fold dilution in VTM, single-use 200 µL aliquots made and frozen at − 80 °C until required. SARS-CoV-2 NAT IS (NIBSC 19/304) was resuspended as per instructions for use and diluted in twelve half-log steps in VTM. Nucleic acid extractions were performed in duplicate on a Roche MagNA Pure 24 extraction platform and from a 50 µL eluate, 5 µL used in triplicate RT-qPCR reactions on an Agilent genetic analyser targeting the E gene using primers, probe and conditions published in Corman et al.,^56^. Viral shedding data was finally expressed as Log_10_ genomic RNA expressed as International Units per mL (IU/mL) calibrated against the WHO RNA International Standard (IS) for SARS-CoV-2 RNA (NIBSC: 20/146) following triplicate regression analysis with comparison of the Vic-01 standard curve analysed in parallel with the WHO IS.

### Subgenomic RNA quantification

ORF-7 subgenomic transcripts were quantified from RNA derived from swabs and faecal pellets using published primer sequences^13^, with triplicate RT-qPCR reactions containing 5 µL extracted RNA quantified against a dilution series of synthesised plasmid (Genewiz) containing 5′UTR and ORF-7 primer and probe targets.

Forward-TCCCAGGTAACAAACCAACC, Reverse-GCTCACAAGTAGCGAGTGTTAT, FAM-ZEN-CTTGTAGATCTGTTCTCTAAACGAAC-IBFQ (probe). Data are expressed as log_10_ subgenomic RNA copies/mL.

### Extraction and quantification of tissue RNA by RT-qPCR

Tissues preserved in RNAlater were subjected to a total RNA extraction protocol. Data are expressed as Log_10_ IU/µg total RNA for each tissue. Tissue was first disrupted and homogenised by bead beating (Qiagen Tissuelyser), strained (BD Biosciences, Filcon, cup type #340631) and total RNA extracted as described above on the automated MagnaPure24 platform (Roche). Total recovered nucleic acid was quantified by spectroscopy and 100 ng loaded per triplicate RT-qPCR reaction as described. House-keeping genes rhGAPDH^57^ or RLP18 RT-PCR^58^ were assayed in parallel to identify possible PCR inhibition. Normalisation of input using Nanodrop and triplicate PCRs were performed on an Agilent genetic analyser with housekeeping gene using RLP 18 (hamster) RLP18F-GTTTATGAGTCGCACTAACCG and RLP18R-TGTTCTCTCGGCCAGGAA with RLP18TM 6-FAM TCTGTCCCTGTCCCGGATGATC BHQ1 probe. GAPDH analysis of NHP samples was applied as previously described^57^ qGAPDH-R GGCTGAGAACGGGAAGCTC; qGAPDH-F AGGGATCTCGCTCCTGGAA and probe rhGAPDH-P 6FAM TCATCAATGGAAGCCCCATCACCA-BHQ1.

### Serology

Competition ELISA, cPass™ SARS-CoV-2 Neutralization Antibody Detection Kit (Genscript, L00847), which detects antibodies that bind competitively to the receptor binding domain (RBD) of SARS-CoV-2 was used according to the manufacturer’s instructions. Where undiluted samples achieved 100% competition eight two-fold serial dilution of the sample were prepared to titrate antibody concentration and obtain a reading within the dynamic range of the assay. Neutralising antibody titres were assessed using a microneutralisation assay (MNA)^59^.

### Virus microculture

Virus culture was assessed in microtiter plate format using VeroE6/TMPRSS2 cells. 20 µL of 1.2 mLs VTM sample was added to duplicate cultures of VeroE6/TMPRSS2 cells (undiluted or 1:2 dilution) in VTM to mitigate interfering or inhibitory/contaminating factors. VeroE6/TMPRSS2 cells seeded into 96 well plates (2 × 10^4^/well/100 µL) were settled overnight prior to washing with PBS and infection for 1 h. 200 µL media was added, cells incubated for 3 days, supernatant removed and cells fixed for 30 min with 4% NBF and stained with 0.5% methyl violet. Cell death was observed, a sample considered positive by culture if at least one of four replicate wells showed destruction of the cell monolayer and presence of SARS-CoV-2 verified by RT-qPCR.

### Rapid antigen (lateral flow) test

BioSensor SARS-CoV-2 Ag point of care kits (Oxford Biosystems) were performed in accordance with manufacturers’ instructions. 350 µL VTM mixed with swabs (nasal or oral) were added to lateral flow devices, the output scored according to the WHO scoring system (https://extranet.who.int/pqweb/vitro-diagnostics/performance-evaluation) and photographed for evidence.

### Immunohistochemistry

Immunohistochemical staining was performed using the Leica Bond RXm automated stainer, Bond Polymer Refine staining system (Leica Microsystems DS9800) and associated Leica Bond consumables. Onboard de-waxing was performed in accordance with the standard Leica Bond protocol and staining undertaken using IHC Protocol F with the following adaptations: additional non-specific block prior to primary antibody incubation (10% normal horse serum (Biorad), 1 × Caesin (Vector Labs) in PBS) and extended haematoxylin staining time (10 min). Antigen unmasking to allow antibody binding was undertaken using optimised conditions for each antibody/antigen combination. Antibodies were diluted to their optimal staining concentration in Bond primary antibody diluent (Leica, AR9352). SARS-CoV-2 Spike protein: 1:1000 HIER1 (Leica, AR9961) 30 min, (Rabbit PAb 40150-T62-COV2-SIB, Stratech Scientific /SinoBiologicals, SARS-CoV-2 Nucleoprotein: 1:2000 HIER 1 (Leica, AR9961) 30 min, (Mouse Mab 40143-MM05-SIB, Stratech Scientific /SinoBiologicals.

### Histopathology analyses and scoring

Four micron thick sections were mounted on poly-l-lysine coated slides (VWR). Prior to manual staining protocols, sections were de-waxed with xylene (Fisher Scientific) and re-hydrated via graded ethanol: water solutions (Fisher Scientific). Lung sections were stained in accordance with standard histological procedures. H&E sections were evaluated for pathological changes associated with disease and assigned a score (0–4; absent, minimal, mild, moderate and marked) for each variable, independently assessed by two board-certified veterinary pathologists blinded to the experimental details.

### RNAscope in situ hybridisation

In situ RNA detection was performed using the RNAscope 2.5 HD manual DAB detection system (Advanced Cell Diagnostics, 322300) and a SARS-CoV-2 specific probe (*V-nCoV2019-S-sense 845701/V-nCoV2019-S 848561)* in accordance with manufacturer’s instructions. Negative (DaPB 310043) and positive (Hs-UBC 310041/Mfa-UBC 461331) control probes were used to assess technique efficiency. Equivalent tissue sections from infection naïve cynomolgus macaques and hamsters were stained with SARS-CoV-2 specific, positive or negative control probes to determine species tissue cross-reactivities. Sections were manually counter stained with haematoxylin.

### Electron microscopy

Samples were prepared for cryosectioning according to the method of Tokuyasu et al.^60^. Briefly, post-mortem biopsies of lung were chemically fixed in 2% formaldehyde and 0.125% glutaraldehyde in PBS for a minimum of 1 h at room temperature and 24 h at 4 °C. Fixed material was re-sectioned to produce ~ 1 mm^3^ cubes on material infiltrated with liquid 12% gelatine in PBS at 37 °C for 36 h with rotation. Tissue cubes were solidified on ice before transfer to cryovials containing 2.3 M sucrose in PBS, transferred to 4 °C with agitation for a minimum of 24 h. Individual cubes were trimmed to remove excess gelatine and mounted onto aluminium specimen pins, excess sucrose solution removed, and the pin and sample plunged into liquid nitrogen. Frozen samples of lung form each species were transferred to a precooled UC6 cryo-microtome at − 120 °C and section of 70–150 nm and cut with a diamond knife (Leica Microsystems (UK) Ltd, Milton Keynes, UK and Diatome, Biel/Bienne, Switzerland respectively). Frozen sections were collected on a 1:1 mixture of 2% methylcellulose and 2.3 M sucrose mounted on carbon-coated copper grids (Agar Scientific, Stanstead, UK) and prepared for imaging. Grids were transferred through a series of droplets of 1% glutaraldehyde in dH_2_O, 1.5% glycine in dH_2_O, dH_2_O before being embedded in a thin layer of 2% methylcellulose with 0.4% uranyl acetate, pH 4.0, and air-dried. Grids prepared for immunolabelling were prepared by transfer through a series of droplets, 1.5% glycine in dH_2_O, then 1% BSA in PBS before incubation with primary antibody for 1 h at room temperature; followed by further BSA washes and incubation with secondary antibody conjugated to 10 nm gold for an additional hour. Grids were washed with dH_2_0, fixed with 1% glutaraldehyde and embedded in thin layer of 2% methylcellulose with 0.4% uranyl acetate, as above. Sections were imaged for electron microscopy in a JEOL JEM 2100 electron microscopes (JEOL (UK) Ltd., Welwyn Garden City, UK) at magnifications ranging from 2000 × to 30,000 ×. Images were captured on a Gatan US4000 CCD camera running Digital micrograph software (Gatan Inc., Pleasanton, CA).

### Statistics

Statistical analyses and graphing were performed using SigmaPlot v12.5. Additional graphing was performed using GraphPad Prism Version 9.3. All images presented in manuscript were generated by co-authors with these two software versions.

## Supplementary Information

Supplementary Information.

## Data Availability

The DNA sequence datasets generated during the current study are available as SRA biosample submissions under entries: SAMN18632196 SARS‐CoV‐2/human/AUS/VIC01/2020 and SAMN18632233 SARS‐CoV‐2/human/AUS/VIC01/2020 at weblinks https://www.ncbi.nlm.nih.gov/biosample/SAMN18632196 SRA and https://www.ncbi.nlm.nih.gov/biosample/SAMN18632233 SRA respectively.

## References

[1] Andersen KG, Rambaut A, Lipkin WI, Holmes EC, Garry RF (2020). The proximal origin of SARS-CoV-2. Nat. Med..

[2] Muñoz-Fontela C, Dowling WE, Funnell SGP, Gsell PS, Riveros-Balta AX, Albrecht RA, Andersen H, Baric RS, Carroll MW, Cavaleri M, Qin C, Crozier I, Dallmeier K, de Waal L, de Wit E, Delang L, Dohm E, Duprex WP, Falzarano D, Finch CL, Frieman MB, Graham BS, Gralinski LE, Guilfoyle K, Haagmans BL, Hamilton GA, Hartman AL, Herfst S, Kaptein SJF, Klimstra WB, Knezevic I, Krause PR, Kuhn JH, Le Grand R, Lewis MG, Liu WC, Maisonnasse P, McElroy AK, Munster V, Oreshkova N, Rasmussen AL, Rocha-Pereira J, Rockx B, Rodríguez E, Rogers TF, Salguero FJ, Schotsaert M, Stittelaar KJ, Thibaut HJ, Tseng CT, Vergara-Alert J, Beer M, Brasel T, Chan JFW, García-Sastre A, Neyts J, Perlman S, Reed DS, Richt JA, Roy CJ, Segalés J, Vasan SS, Henao-Restrepo AM, Barouch DH (2020). Animal models for COVID-19. Nature.

[3] Callender LA, Curran M, Bates SM, Mairesse M, Weigandt J, Betts CJ (2020). The impact of pre-existing comorbidities and therapeutic interventions on COVID-19. Front. Immunol..

[4] Tao K, Tzou PL, Nouhin J, Gupta RK, de Oliveira T, Kosakovsky Pond SL, Fera D, Shafer RW (2021). The biological and clinical significance of emerging SARS-CoV-2 variants. Nat. Rev. Genet..

[5] Damas J, Hughes GM, Keough KC, Painter CA, Persky NS, Corbo M, Hiller M, Koepfli KP, Pfenning AR, Zhao H, Genereux DP, Swofford R, Pollard KS, Ryder OA, Nweeia MT, Lindblad-Toh K, Teeling EC, Karlsson EK, Lewin HA (2020). Broad host range of SARS-CoV-2 predicted by comparative and structural analysis of ACE-2 in vertebrates. Proc. Natl. Acad. Sci. USA..

[6] Liu Y, Hu G, Wang Y, Ren W, Zhao X, Ji F, Zhu Y, Feng F, Gong M, Ju X, Zhu Y, Cai X, Lan J, Guo J, Xie M, Dong L, Zhu Z, Na J, Wu J, Lan X, Xie Y, Wang X, Yuan Z, Zhang R, Ding Q (2021). Functional and genetic analysis of viral receptor ACE2 orthologs reveals a broad potential host range of SARS-CoV-2. Proc. Natl. Acad. Sci. USA..

[7] Zhao X, Chen D, Szabla R, Zheng M, Li G, Du P, Zheng S, Li X, Song C, Li R, Guo JT, Junop M, Zeng H, Lin H (2020). Broad and differential animal angiotensin-converting enzyme 2 receptor usage by SARS-CoV-2. J. Virol..

[8] Shi J, Wen Z, Zhong G, Yang H, Wang C, Huang B, Liu R, He X, Shuai L, Sun Z, Zhao Y, Liu P, Liang L, Cui P, Wang J, Zhang X, Guan Y, Tan W, Wu G, Chen H, Bu Z (2020). Susceptibility of ferrets, cats, dogs, and other domesticated animals to SARS-coronavirus 2. Science.

[9] Conceicao C, Thakur N, Human S, Kelly JT, Logan L, Bialy D, Bhat S, Stevenson-Leggett P, Zagrajek AK, Hollinghurst P, Varga M, Tsirigoti C, Tully M, Chiu C, Moffat K, Silesian AP, Hammond JA, Maier HJ, Bickerton E, Shelton H, Dietrich I, Graham SC, Bailey D (2020). The SARS-CoV-2 Spike protein has a broad tropism for mammalian ACE2 proteins. PLoS Biol..

[10] Rockx B, Kuiken T, Herfst S, Bestebroer T, Lamers MM, OudeMunnink BB, de Meulder D, van Amerongen G, van den Brand J, Okba NMA, Schipper D, van Run P, Leijten L, Sikkema R, Verschoor E, Verstrepen B, Bogers W, Langermans J, Drosten C, van Vlissingen MF, Fouchier R, de Swart R, Koopmans M, Haagmans BL (2020). Comparative pathogenesis of COVID-19, MERS, and SARS in a nonhuman primate model. Science.

[11] Koo BS, Oh H, Kim G, Hwang EH, Jung H, Lee Y, Kang P, Park JH, Ryu CM, Hong JJ (2020). Transient lymphopenia and interstitial pneumonia with endotheliitis in SARS-CoV-2-infected macaques. J. Infect. Dis..

[12] Salguero FJ, White AD, Slack GS, Fotheringham SA, Bewley KR, Gooch KE, Longet S, Humphries HE, Watson RJ, Hunter L, Ryan KA, Hall Y, Sibley L, Sarfas C, Allen L, Aram M, Brunt E, Brown P, Buttigieg KR, Cavell BE, Cobb R, Coombes NS, Darby A, Daykin-Pont O, Elmore MJ, Garcia-Dorival I, Gkolfinos K, Godwin KJ, Gouriet J, Halkerston R, Harris DJ, Hender T, Ho CMK, Kennard CL, Knott D, Leung S, Lucas V, Mabbutt A, Morrison AL, Nelson C, Ngabo D, Paterson J, Penn EJ, Pullan S, Taylor I, Tipton T, Thomas S, Tree JA, Turner C, Vamos E, Wand N, Wiblin NR, Charlton S, Dong X, Hallis B, Pearson G, Rayner EL, Nicholson AG, Funnell SG, Hiscox JA, Dennis MJ, Gleeson FV, Sharpe S, Carroll MW (2021). Comparison of rhesus and cynomolgus macaques as an infection model for COVID-19. Nat. Commun..

[13] Munster VJ, Feldmann F, Williamson BN, van Doremalen N, Pérez-Pérez L, Schulz J, Meade-White K, Okumura A, Callison J, Brumbaugh B, Avanzato VA, Rosenke R, Hanley PW, Saturday G, Scott D, Fischer ER, de Wit E (2020). Respiratory disease in rhesus macaques inoculated with SARS-CoV-2. Nature.

[14] Singh DK, Singh B, Ganatra SR, Gazi M, Cole J, Thippeshappa R, Alfson KJ, Clemmons E, Gonzalez O, Escobedo R, Lee TH, Chatterjee A, Goez-Gazi Y, Sharan R, Gough M, Alvarez C, Blakley A, Ferdin J, Bartley C, Staples H, Parodi L, Callery J, Mannino A, Klaffke B, Escareno P, Platt RN, Hodara V, Scordo J, Gautam S, Vilanova AG, Olmo-Fontanez A, Schami A, Oyejide A, Ajithdoss DK, Copin R, Baum A, Kyratsous C, Alvarez X, Ahmed M, Rosa B, Goodroe A, Dutton J, Hall-Ursone S, Frost PA, Voges AK, Ross CN, Sayers K, Chen C, Hallam C, Khader SA, Mitreva M, Anderson TJC, Martinez-Sobrido L, Patterson JL, Turner J, Torrelles JB, Dick EJ, Brasky K, Schlesinger LS, Giavedoni LD, Carrion R, Kaushal D (2021). Responses to acute infection with SARS-CoV-2 in the lungs of rhesus macaques, baboons and marmosets. Nat. Microbiol..

[15] Hartman AL, Nambulli S, McMillen CM, White AG, Tilston-Lunel NL, Albe JR, Cottle E, Dunn MD, Frye LJ, Gilliland TH, Olsen EL, O'Malley KJ, Schwarz MM, Tomko JA, Walker RC, Xia M, Hartman MS, Klein E, Scanga CA, Flynn JL, Klimstra WB, McElroy AK, Reed DS, Duprex WP (2020). SARS-CoV-2 infection of African green monkeys results in mild respiratory disease discernible by PET/CT imaging and shedding of infectious virus from both respiratory and gastrointestinal tracts. PLoS Pathog..

[16] Chandrashekar A, Liu J, Martinot AJ, McMahan K, Mercado NB, Peter L, Tostanoski LH, Yu J, Maliga Z, Nekorchuk M, Busman-Sahay K, Terry M, Wrijil LM, Ducat S, Martinez DR, Atyeo C, Fischinger S, Burke JS, Slein MD, Pessaint L, Van Ry A, Greenhouse J, Taylor T, Blade K, Cook A, Finneyfrock B, Brown R, Teow E, Velasco J, Zahn R, Wegmann F, Abbink P, Bondzie EA, Dagotto G, Gebre MS, He X, Jacob-Dolan C, Kordana N, Li Z, Lifton MA, Mahrokhian SH, Maxfield LF, Nityanandam R, Nkolola JP, Schmidt AG, Miller AD, Baric RS, Alter G, Sorger PK, Estes JD, Andersen H, Lewis MG, Barouch DH (2020). SARS-CoV-2 infection protects against rechallenge in rhesus macaques. Science.

[17] Imai M, Iwatsuki-Horimoto K, Hatta M, Loeber S, Halfmann PJ, Nakajima N, Watanabe T, Ujie M, Takahashi K, Ito M, Yamada S, Fan S, Chiba S, Kuroda M, Guan L, Takada K, Armbrust T, Balogh A, Furusawa Y, Okuda M, Ueki H, Yasuhara A, Sakai-Tagawa Y, Lopes TJS, Kiso M, Yamayoshi S, Kinoshita N, Ohmagari N, Hattori SI, Takeda M, Mitsuya H, Krammer F, Suzuki T, Kawaoka Y (2020). Syrian hamsters as a small animal model for SARS-CoV-2 infection and countermeasure development. Proc. Natl. Acad. Sci..

[18] Sia SF, Yan LM, Chin AWH, Fung K, Choy KT, Wong AYL, Kaewpreedee P, Perera RAPM, Poon LLM, Nicholls JM, Peiris M, Yen HL (2020). Pathogenesis and transmission of SARS-CoV-2 in golden hamsters. Nature.

[19] Lee AC, Zhang AJ, Chan JF, Li C, Fan Z, Liu F, Chen Y, Liang R, Sridhar S, Cai JP, Poon VK, Chan CC, To KK, Yuan S, Zhou J, Chu H, Yuen KY (2020). Oral SARS-CoV-2 inoculation establishes subclinical respiratory infection with virus shedding in golden Syrian hamsters. Cell Rep. Med..

[20] Tostanoski LH, Wegmann F, Martinot AJ, Loos C, McMahan K, Mercado NB, Yu J, Chan CN, Bondoc S, Starke CE, Nekorchuk M, Busman-Sahay K, Piedra-Mora C, Wrijil LM, Ducat S, Custers J, Atyeo C, Fischinger S, Burke JS, Feldman J, Hauser BM, Caradonna TM, Bondzie EA, Dagotto G, Gebre MS, Jacob-Dolan C, Lin Z, Mahrokhian SH, Nampanya F, Nityanandam R, Pessaint L, Porto M, Ali V, Benetiene D, Tevi K, Andersen H, Lewis MG, Schmidt AG, Lauffenburger DA, Alter G, Estes JD, Schuitemaker H, Zahn R, Barouch DH (2020). Ad26 vaccine protects against SARS-CoV-2 severe clinical disease in hamsters. Nat. Med..

[21] Chan JF, Zhang AJ, Yuan S, Poon VK, Chan CC, Lee AC, Chan WM, Fan Z, Tsoi HW, Wen L, Liang R, Cao J, Chen Y, Tang K, Luo C, Cai JP, Kok KH, Chu H, Chan KH, Sridhar S, Chen Z, Chen H, To KK, Yuen KY (2020). Simulation of the clinical and pathological manifestations of coronavirus disease 2019 (COVID-19) in a golden Syrian hamster model: Implications for disease pathogenesis and transmissibility. Clin. Infect. Dis..

[22] Zhang AJ, Lee AC, Chu H, Chan JF, Fan Z, Li C, Liu F, Chen Y, Yuan S, Poon VK, Chan CC, Cai JP, Wu KL, Sridhar S, Chan YS, Yuen KY (2020). SARS-CoV-2 infects and damages the mature and immature olfactory sensory neurons of hamsters. Clin. Infect. Dis..

[23] Brocato RL, Principe LM, Kim RK, Zeng X, Williams JA, Liu Y, Li R, Smith JM, Golden JW, Gangemi D, Youssef S, Wang Z, Glanville J, Hooper JW (2020). Disruption of adaptive immunity enhances disease in SARS-CoV-2-infected Syrian hamsters. J. Virol..

[24] Berry N, Ferguson D, Ham C, Hall J, Jenkins A, Giles E, Kempster S, Rose N, Dowall S, Fritzsche M, Bleazard T, Hewson R, Almond N (2019). High susceptibility, viral dynamics and persistence of South American Zika virus in New World monkey species. Sci. Rep..

[25] Berry N, Kempster S, Ham C, Jenkins A, Hall J, Page M, Mattiuzzo G, Giles E, Ferguson D, Almond N (2020). Passive immunisation of convalescent human anti-Zika serum protects against challenge with New World Zika virus in cynomolgus macaques. NPJ Vaccines.

[26] Funnell SGP, Afrough B, Baczenas JJ, Berry N, Bewley KR, Bradford R, Florence C, Duff YL, Lewis M, Moriarty RV, Connor SLO, Osman KL, Pullan S, Rashid S, Richards KS, Stemple KJ, Knezevic I (2021). A cautionary perspective regarding the isolation and serial propagation of SARS-CoV-2 in Vero cells. NPJ Vaccines..

[27] Fears AC, Klimstra WB, Duprex P, Hartman A, Weaver SC, Plante KS, Mirchandani D, Plante JA, Aguilar PV, Fernández D, Nalca A, Totura A, Dyer D, Kearney B, Lackemeyer M, Bohannon JK, Johnson R, Garry RF, Reed DS, Roy CJ (2020). Persistence of severe acute respiratory syndrome coronavirus 2 in aerosol suspensions. Emerg. Infect. Dis..

[28] Dabisch PA, Biryukov J, Beck K, Boydston JA, Sanjak JS, Herzog A, Green B, Williams G, Yeager J, Bohannon JK, Holland B, Miller D, Reese AL, Freeburger D, Miller S, Jenkins T, Rippeon S, Miller J, Clarke D, Manan E, Patty A, Rhodes K, Sweeney T, Winpigler M, Price O, Rodriguez J, Altamura LA, Zimmerman H, Hail AS, Wahl V, Hevey M (2021). Seroconversion and fever are dose-dependent in a nonhuman primate model of inhalational COVID-19. PLoS Pathog..

[29] Blair RV, Vaccari M, Doyle-Meyers LA, Roy CJ, Russell-Lodrigue K, Fahlberg M, Monjure CJ, Beddingfield B, Plante KS, Plante JA, Weaver SC, Qin X, Midkiff CC, Lehmicke G, Golden N, Threeton B, Penney T, Allers C, Barnes MB, Pattison M, Datta PK, Maness NJ, Birnbaum A, Fischer T, Bohm RP, Rappaport J (2021). Acute respiratory distress in aged, SARS-CoV-2-infected African green monkeys but not rhesus macaques. Am. J. Pathol..

[30] Brosseau LM, Escandón K, Ulrich AK, Rasmussen AL, Roy CJ, Bix GJ, Popescu SV, Moore K, Osterholm MT (2021). SARS-CoV-2 dose, infection, and disease outcomes for COVID-19: A review. Clin. Infect. Dis..

[31] Edwards DA, Ausiello D, Salzman J, Devlin T, Langer R, Beddingfield BJ, Fears AC, Doyle-Meyers LA, Redmann RK, Killeen SZ, Maness NJ, Roy CJ (2021). Exhaled aerosol increases with COVID-19 infection, age, and obesity. Proc. Natl. Acad. Sci. USA.

[32] O’Connor SL, Lhost JJ, Becker EA, Detmer AM, Johnson RC, Macnair CE, Wiseman RW, Karl JA, Greene J, Burwitz BB, Bimber BN, Lank SM, Tuscher JT, Mee ET, Rose NJ, Desrosiers RC, Hughes AL, Friedrich TC, Carrington M, O’Connor DH (2010). MHC heterozygote advantage in simian immunodeficiency virus-infected Mauritian cynomolgus macaques. Sci. Transl. Med..

[33] Swadling L, Diniz MO, Schmidt NM, Amin OE, Chandran A, Shaw E, Pade C, Gibbons JM, LeBert N, Tan AT, Jeffery-Smith A, Tan CCS, Tham CYL, Kucykowicz S, Aidoo-Micah G, Rosenheim J, Davies J, Johnson M, Jensen MP, Joy G, McCoy LE, Valdes AM, Chain BM, Goldblatt D, Altmann DM, Boyton RJ, Manisty C, Treibel TA, Moon JC, Dorp LV, Balloux F, McKnight Á, Noursadeghi M, Bertoletti A, Maini MK (2021). Pre-existing polymerase-specific T cells expand in abortive seronegative SARS-CoV-2. Nature.

[34] Meekins DA, Morozov I, Trujillo JD, Gaudreault NN, Bold D, Carossino M, Artiaga BL, Indran SV, Kwon T, Balaraman V, Madden DW, Feldmann H, Henningson J, Ma W, Balasuriya UB, Richt JA (2020). Susceptibility of swine cells and domestic pigs to SARS-CoV-2. Emerg. Microbes Infect..

[35] Gaudreault NN, Trujillo JD, Carossino M, Meekins DA, Morozov I, Madden DW, Indran SV, Bold D, Balaraman V, Kwon T, Artiaga BL, Cool K, García-Sastre A, Ma W, Wilson WC, Henningson J, Balasuriya UBR, Richt JA (2020). SARS-CoV-2 infection, disease and transmission in domestic cats. Emerg. Microbes Infect..

[36] Schlottau K, Rissmann M, Graaf A, Schön J, Sehl J, Wylezich C, Höper D, Mettenleiter TC, Balkema-Buschmann A, Harder T, Grund C, Hoffmann D, Breithaupt A, Beer M (2020). SARS-CoV-2 in fruit bats, ferrets, pigs, and chickens: An experimental transmission study. Lancet Microbe.

[37] Lu S, Zhao Y, Yu W, Yang Y, Gao J, Wang J, Kuang D, Yang M, Yang J, Ma C, Xu J, Qian X, Li H, Zhao S, Li J, Wang H, Long H, Zhou J, Luo F, Ding K, Wu D, Zhang Y, Dong Y, Liu Y, Zheng Y, Lin X, Jiao L, Zheng H, Dai Q, Sun Q, Hu Y, Ke C, Liu H, Peng X (2020). Comparison of nonhuman primates identified the suitable model for COVID-19. Signal Transduct. Target Ther..

[38] Buss LF, Prete CA, Abrahim CMM, Mendrone A, Salomon T, de Almeida-Neto C, França RFO, Belotti MC, Carvalho MPSS, Costa AG, Crispim MAE, Ferreira SC, Fraiji NA, Gurzenda S, Whittaker C, Kamaura LT, Takecian PL, da Silva PP, Oikawa MK, Nishiya AS, Rocha V, Salles NA, de Souza Santos AA, da Silva MA, Custer B, Parag KV, Barral-Netto M, Kraemer MUG, Pereira RHM, Pybus OG, Busch MP, Castro MC, Dye C, Nascimento VH, Faria NR, Sabino EC (2021). Three-quarters attack rate of SARS-CoV-2 in the Brazilian Amazon during a largely unmitigated epidemic. Science.

[39] Xavier J, Giovanetti M, Adelino T, Fonseca V, da Costa AVB, Ribeiro AA, Felicio KN, Duarte CG, Ferreira Silva MV, Salgado Á, Lima MT, de Jesus R, Fabri A, Soares Zoboli CF, Souza Santos TG, Iani F, Ciccozzi M, de Filippis AMB, de Siqueira MAMT, de Abreu AL, de Azevedo V, Ramalho DB, de Albuquerque CFC, de Oliveira T, Holmes EC, Lourenço J, Junior Alcantara LC, AssunçãoOliveira MA (2020). The ongoing COVID-19 epidemic in Minas Gerais, Brazil: insights from epidemiological data and SARS-CoV-2 whole genome sequencing. Emerg. Microbes Infect..

[40] Palmer MV, Martins M, Falkenberg S, Buckley A, Caserta LC, Mitchell PK, Cassmann ED, Rollins A, Zylich NC, Renshaw RW, Guarino C, Wagner B, Lager K, Diel DG (2021). Susceptibility of white-tailed deer (*Odocoileus virginianus*) to SARS-CoV-2. J. Virol..

[41] Fagre A, Lewis J, Eckley M, Zhan S, Rocha SM, Sexton NR, Burke B, Geiss B, Peersen O, Bass T, Kading R, Rovnak J, Ebel GD, Tjalkens RB, Aboellail T, Schountz T (2021). SARS-CoV-2 infection, neuropathogenesis and transmission among deer mice: Implications for spillback to New World rodents. PLoS Pathog..

[42] Griffin BD, Chan M, Tailor N, Mendoza EJ, Leung A, Warner BM, Duggan AT, Moffat E, He S, Garnett L, Tran KN, Banadyga L, Albietz A, Tierney K, Audet J, Bello A, Vendramelli R, Boese AS, Fernando L, Lindsay LR, Jardine CM, Wood H, Poliquin G, Strong JE, Drebot M, Safronetz D, Embury-Hyatt C, Kobasa D (2021). SARS-CoV-2 infection and transmission in the North American deer mouse. Nat. Commun..

[43] Oude Munnink BB, Sikkema RS, Nieuwenhuijse DF, Molenaar RJ, Munger E, Molenkamp R, van der Spek A, Tolsma P, Rietveld A, Brouwer M, Bouwmeester-Vincken N, Harders F, Hakze-vanderHoning R, Wegdam-Blans MCA, Bouwstra RJ, GeurtsvanKessel C, van der Eijk AA, Velkers FC, Smit LAM, Stegeman A, van der Poel WHM, Koopmans MPG (2021). Transmission of SARS-CoV-2 on mink farms between humans and mink and back to humans. Science.

[44] Hammer AS, Quaade ML, Rasmussen TB, Fonager J, Rasmussen M, Mundbjerg K, Lohse L, Strandbygaard B, Jørgensen CS, Alfaro-Núñez A, Rosenstierne MW, Boklund A, Halasa T, Fomsgaard A, Belsham GJ, Bøtner A (2021). SARS-CoV-2 transmission between mink (*Neovison vison*) and Humans, Denmark. Emerg. Infect. Dis..

[45] Rosenke K, Meade-White K, Letko M, Clancy C, Hansen F, Liu Y, Okumura A, Tang-Huau TL, Li R, Saturday G, Feldmann F, Scott D, Wang Z, Munster V, Jarvis MA, Feldmann H (2020). Defining the Syrian hamster as a highly susceptible preclinical model for SARS-CoV-2 infection. Emerg. Microbes Infect..

[46] Ulasli M, Verheije MH, de Haan CA, Reggiori F (2010). Qualitative and quantitative ultrastructural analysis of the membrane rearrangements induced by coronavirus. Cell Microbiol..

[47] Snijder EJ, Limpens RWAL, de Wilde AH, de Jong AWM, Zevenhoven-Dobbe JC, Maier HJ, Faas FFGA, Koster AJ, Bárcena M (2020). A unifying structural and functional model of the coronavirus replication organelle: Tracking down RNA synthesis. PLoS Biol..

[48] Neil D, Moran L, Horsfield C, Curtis E, Swann O, Barclay W, Hanley B, Hollinshead M, Roufosse C (2020). Ultrastructure of cell trafficking pathways and coronavirus: how to recognise the wolf amongst the sheep. J. Pathol..

[49] Nunn AV, Guy GW, Brysch W, Botchway SW, Frasch W, Calabrese EJ, Bell JD (2020). SARS-CoV-2 and mitochondrial health: Implications of lifestyle and ageing. Immunity Ageing.

[50] Corbett KS, Nason MC, Flach B, Gagne M, O’Connell S, Johnston TS, Shah SN, Edara VV, Floyd K, Lai L, McDanal C, Francica JR, Flynn B, Wu K, Choi A, Koch M, Abiona OM, Werner AP, Moliva JI, Andrew SF, Donaldson MM, Fintzi J, Flebbe DR, Lamb E, Noe AT, Nurmukhambetova ST, Provost SJ, Cook A, Dodson A, Faudree A, Greenhouse J, Kar S, Pessaint L, Porto M, Steingrebe K, Valentin D, Zouantcha S, Bock KW, Minai M, Nagata BM, van de Wetering R, Boyoglu-Barnum S, Leung K, Shi W, Yang ES, Zhang Y, Todd JM, Wang L, Alvarado GS, Andersen H, Foulds KE, Edwards DK, Mascola JR, Moore IN, Lewis MG, Carfi A, Montefiori D, Suthar MS, McDermott A, Roederer M, Sullivan NJ, Douek DC, Graham BS, Seder RA (2021). Immune correlates of protection by mRNA-1273 vaccine against SARS-CoV-2 in nonhuman primates. Science.

[51] Dagotto G, Mercado NB, Martinez DR, Hou YJ, Nkolola JP, Carnahan RH, Crowe JE, Baric RS, Barouch DH (2021). Comparison of subgenomic and total RNA in SARS-CoV-2 challenged rhesus macaques. J. Virol..

[52] Foladori P, Cutrupi F, Segata N, Manara S, Pinto F, Malpei F, Bruni L, La Rosa G (2020). SARS-CoV-2 from faeces to wastewater treatment: What do we know? A review. Sci. Total Environ..

[53] Moore SC, Penrice-Randal R, Alruwaili M, Randle N, Armstrong S, Hartley C, Haldenby S, Dong X, Alrezaihi A, Almsaud M, Bentley E, Clark J, García-Dorival I, Gilmore P, Han X, Jones B, Luu L, Sharma P, Shawli G, Sun Y, Zhao Q, Pullan ST, Carter DP, Bewley K, Dunning J, Zhou EM, Solomon T, Beadsworth M, Cruise J, Crook DW, Matthews DA, Davidson AD, Mahmood Z, Aljabr W, Druce J, Vipond R, Ng L, Renia L, Openshaw PJM, Baillie JK, Carroll MW, Stewart J, Darby A, Semple M, Turtle L, Hiscox JA (2020). Amplicon-based detection and sequencing of SARS-CoV-2 in nasopharyngeal swabs from patients with COVID-19 and identification of deletions in the viral genome that encode proteins involved in interferon antagonism. Viruses.

[54] Tyson JR, James P, Stoddart D, Sparks N, Wickenhagen A, Hall G, Choi JH, Lapointe H, Kamelian K, Smith AD, Prystajecky N, Goodfellow I, Wilson SJ, Harrigan R, Snutch TP, Loman NJ, Quick J (2020). Improvements to the ARTIC multiplex PCR method for SARS-CoV-2 genome sequencing using nanopore. BioRxiv.

[55] Castellano S, Cestari F, Faglioni G, Tenedini E, Marino M, Artuso L, Manfredini R, Luppi M, Trenti T, Tagliafico E (2021). iVar, an interpretation-oriented tool to manage the update and revision of variant annotation and classification. Genes.

[56] Corman VM, Landt O, Kaiser M (2020). Detection of 2019 novel coronavirus (2019-nCoV) by real-time RT-PCR. Euro Surveill..

[57] Mattiuzzo G, Rose NJ, Almond N, Towers GJ, Berry N (2013). Upregulation of TRIM5α gene expression after live-attenuated simian immunodeficiency virus vaccination in Mauritian cynomolgus macaques, but TRIM5α genotype has no impact on virus acquisition or vaccination outcome. J. Gen. Virol..

[58] Zivcec M, Safronetz D, Haddock E, Feldmann H, Ebihara H (2011). Validation of assays to monitor immune responses in the Syrian golden hamster (*Mesocricetus auratus*). J. Immunol. Methods.

[59] Bewley KR, Coombes NS, Gagnon L, McInroy L, Baker N, Shaik I, St-Jean JR, St-Amant N, Buttigieg KR, Humphries HE, Godwin KJ, Brunt E, Allen L, Leung S, Brown PJ, Penn EJ, Thomas K, Kulnis G, Hallis B, Carroll M, Funnell S, Charlton S (2021). Quantification of SARS-CoV-2 neutralizing antibody by wild-type plaque reduction neutralization, microneutralization and pseudotyped virus neutralization assays. Nat. Protoc..

[60] Tokuyasu KT (1973). A technique for ultramicrotomy of cell suspensions and tissues. J. Cell. Biol..

