# Supplementary material for: Intrinsic host susceptibility among multiple species to intranasal SARS-CoV-2 identifies diverse virological, biodistribution and pathological outcomes
Source: Sci Rep. 2022 Nov 4;12:18694. doi: 10.1038/s41598-022-23339-x (PMC9636276; doi:10.1038/s41598-022-23339-x)
Supplement: Supplementary file 1 — Supplementary Information. [file 41598_2022_23339_MOESM1_ESM.docx]

**Supplementary Figures**


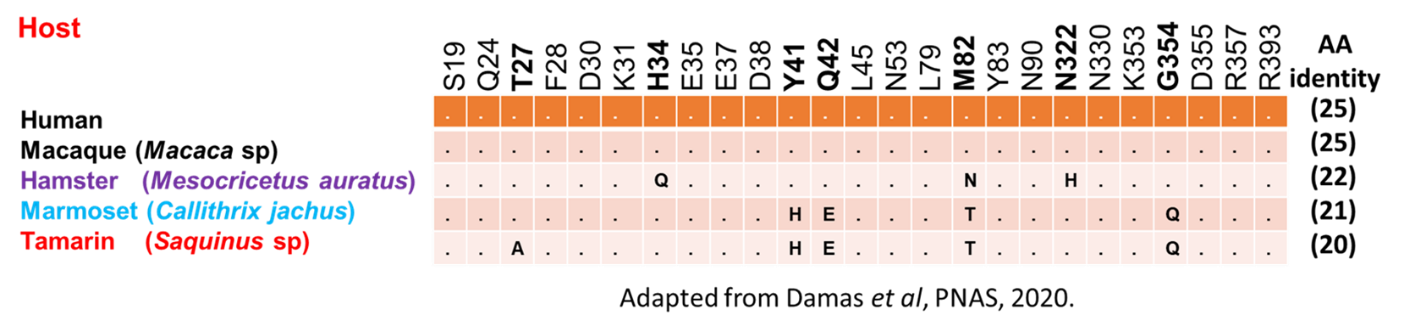


Figure S1. ACE-2 sequence homology of key amino acid residues across the receptor binding domain (RBD). Comparative alignment for key amino acid residues across the receptor binding domain with residue changes from the human consensus indicated for Syrian hamsters, *Macaca* sp, common marmosets and Emperor tamarin (Adapted from Damas *et al,* 2020, ref *5*).


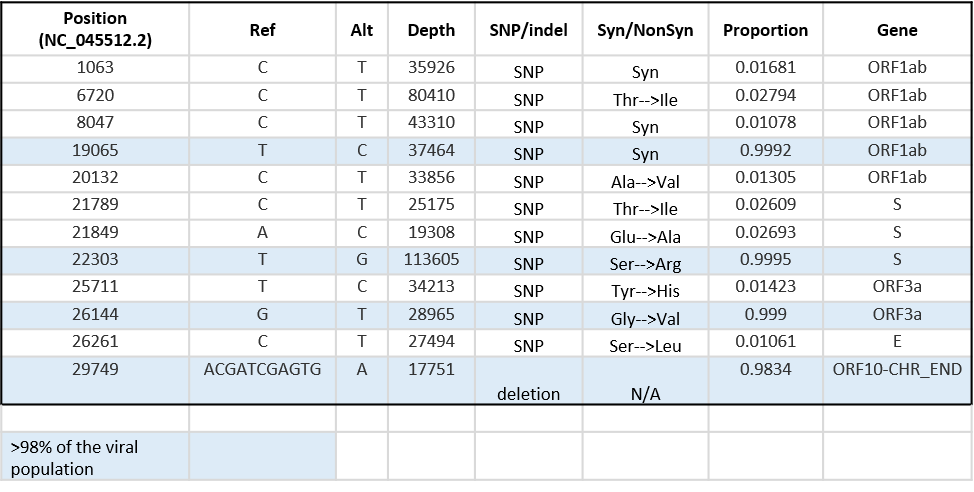


**A)**


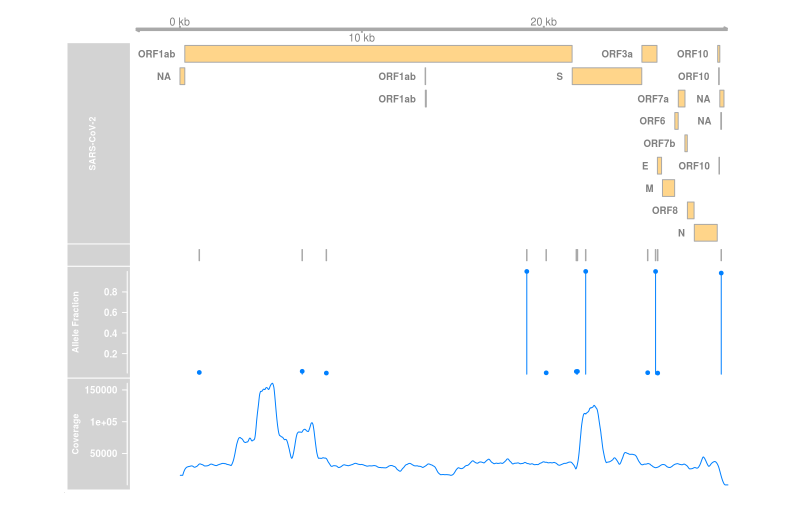


**B)**

Figure S2. Summary of SNP calling of Victoria-01 against Wuhan-01 reference.

A) Coverage depth as indicated with changes in the genes as indicated. B) Pictorial representation of sequence changes across the genome and coverage.


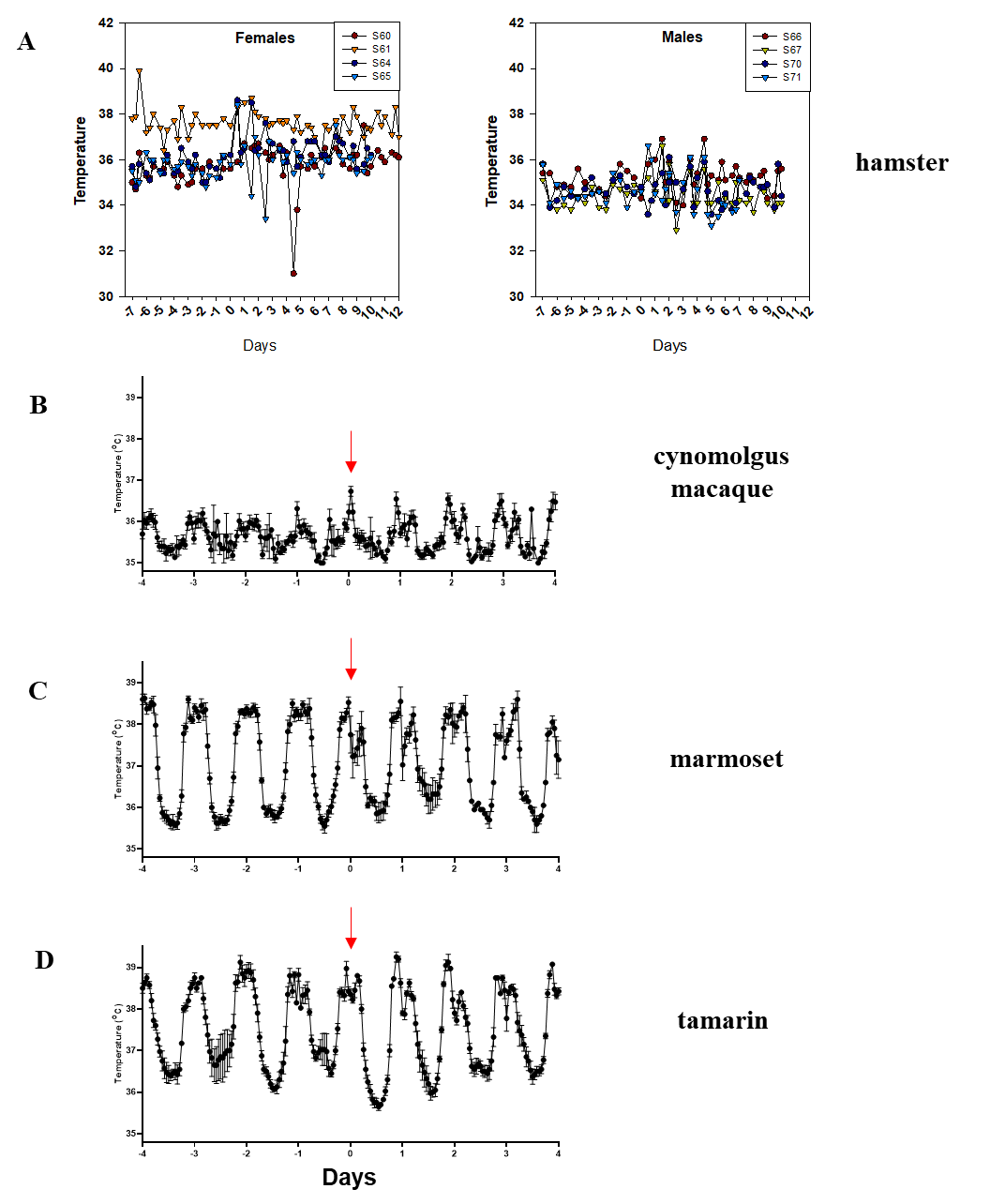


**Figure S3.** **Temperature perturbations following SARS-CoV-2 infection.** A) Diurnal temperature fluctuations in female and male hamsters using remote subcutaneous transplanted transponders 7 days prior to challenge (baseline) and 12 days post-challenge. B) cynomolgus macaques C) marmosets and D) red-bellied tamarins. Individual temperature fluctuations at each reading are indicated by error bars.


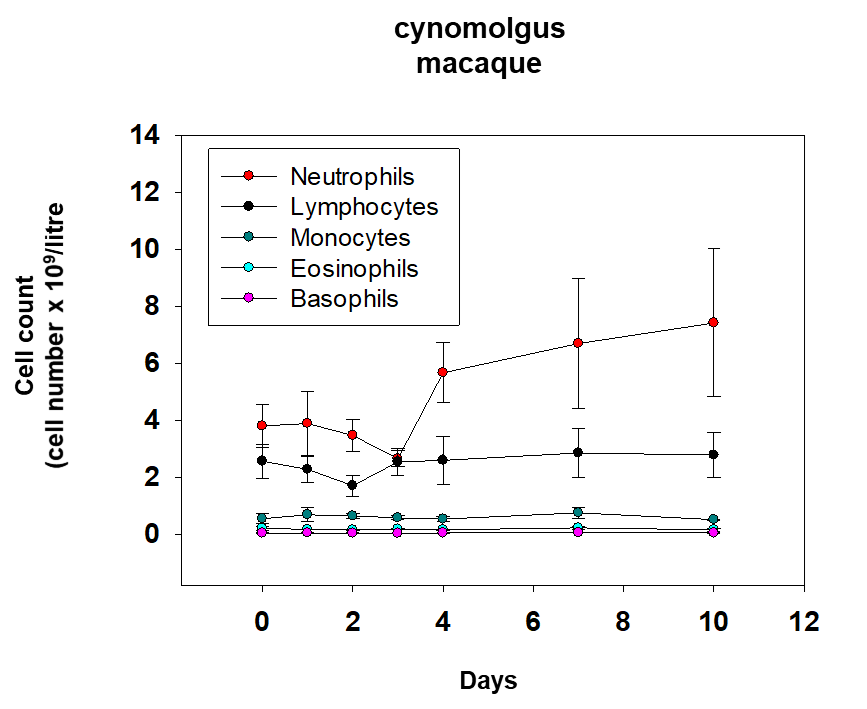


**Figure S4. White cell parameters in cynomolgus macaques over 10 days infection (*n*=4).** Baseline values are represented from day 0 for each marker.

**
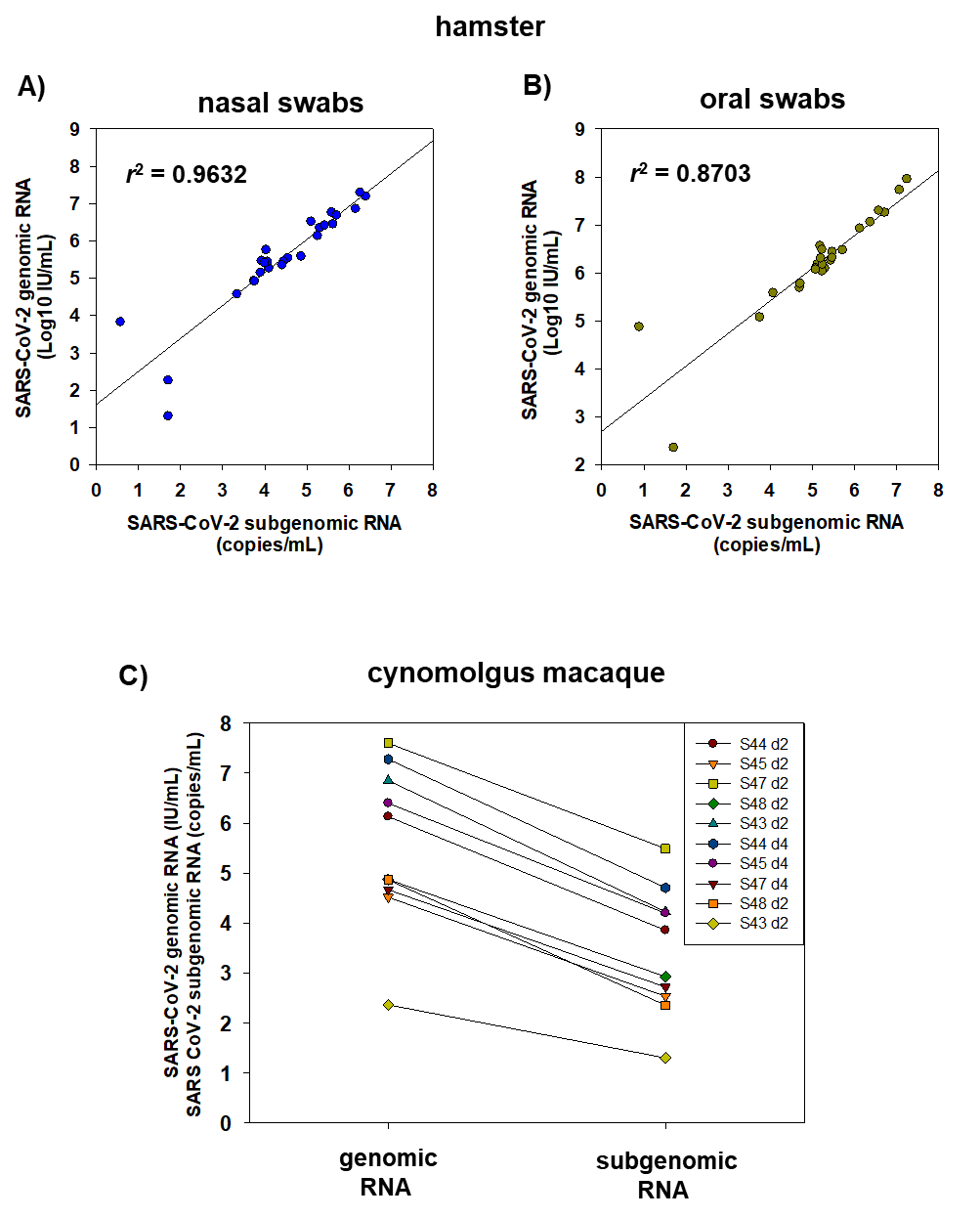
**

**Figure S5**. **Relationship between genomic and subgenomic RNA.** Data are expressed as Log_10_ genomic RNA in International Units (IU)/mL and sub-genomic RNA in copies/mL respectively. A total of 51 individual A) nasal (n=26) and B) oral (n=25) swabs analysed for SARS-CoV-2 genomic (E gene) and subgenomic RNA (Orf-7a gene) targets from hamsters challenged with Vic-01 over the first 7 days of infection. C) Relationship between genomic and sub-genomic RNA in oral swabs from cynomolgus macaques (taken at days 2 and 4 post-infection, *n* =10).


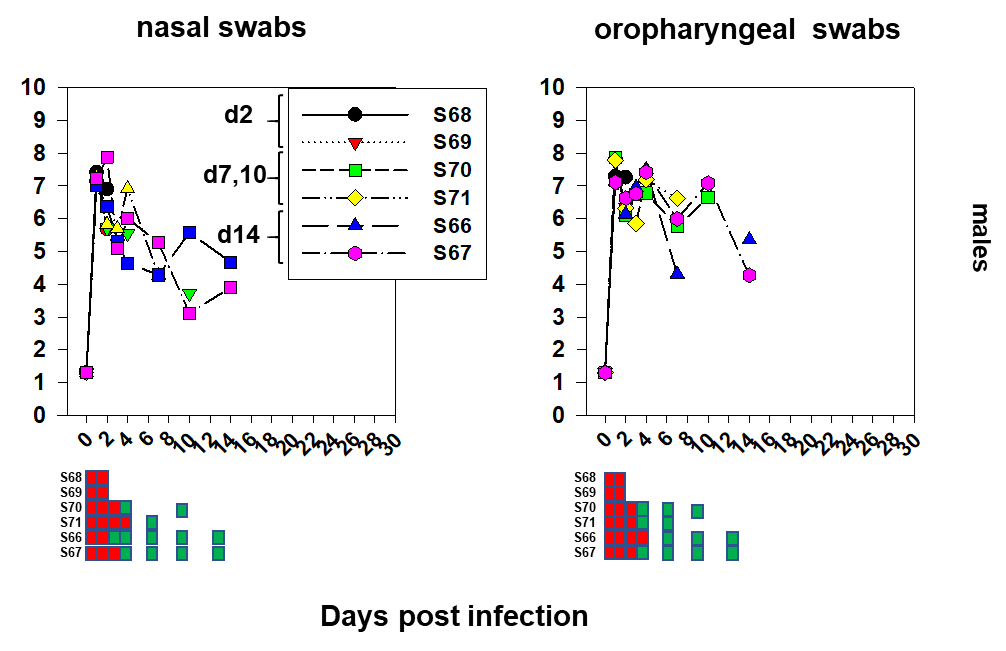


**Figure S6.** **Viral gRNA levels and culture data in male hamsters from nasal and oral swabs.** Male hamsters S70 and S71 (original day 10), terminated at 7 and 10dpi respectively; S66 and S67 (original day 28) euthanased 14dpi.

**Figure S7.** **Genomic RNA levels in rectal swabs from cynomolgus macaques.** Transient increase in gRNA signal in rectal swabs was not associated with recovery of infectious virus in cynomolgus macaques. Green bar indicates virus recovery attempted but unsuccessful on VeroE6/TMPRSS2 cells at the same time-points as swab sampling despite a small rebound in gRNA signal in macaque S46.


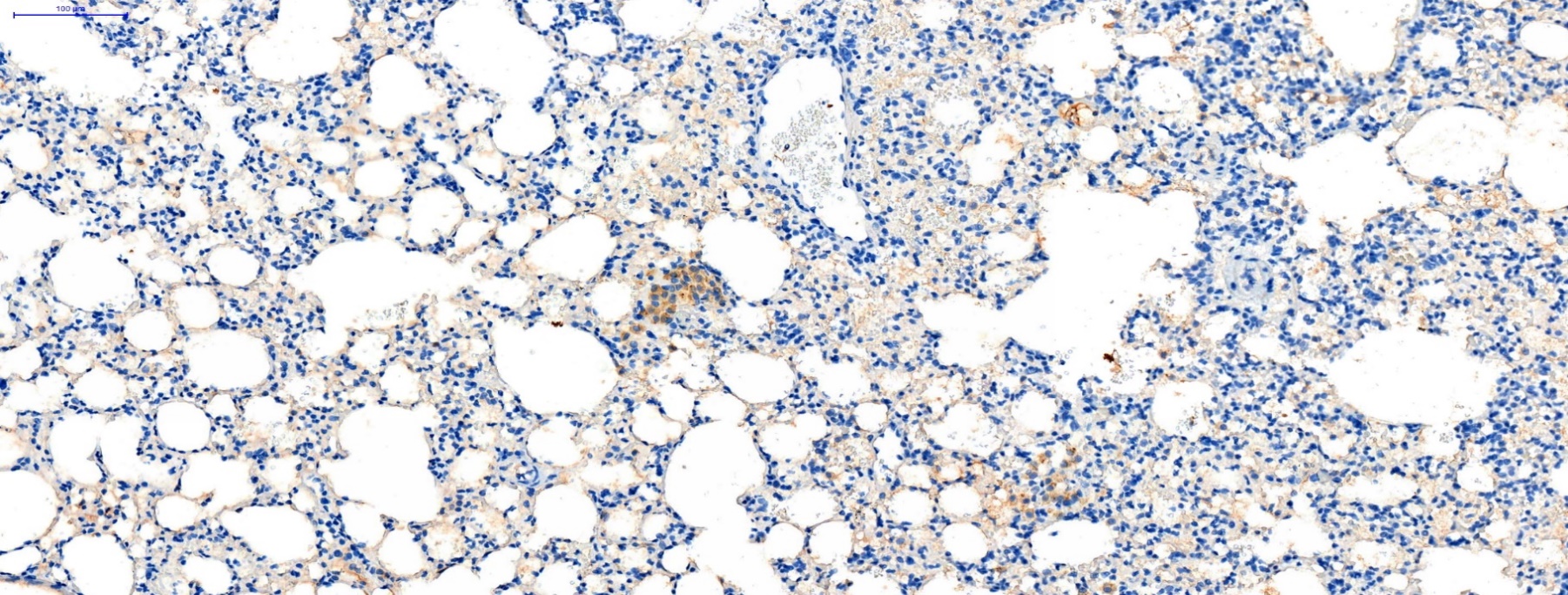

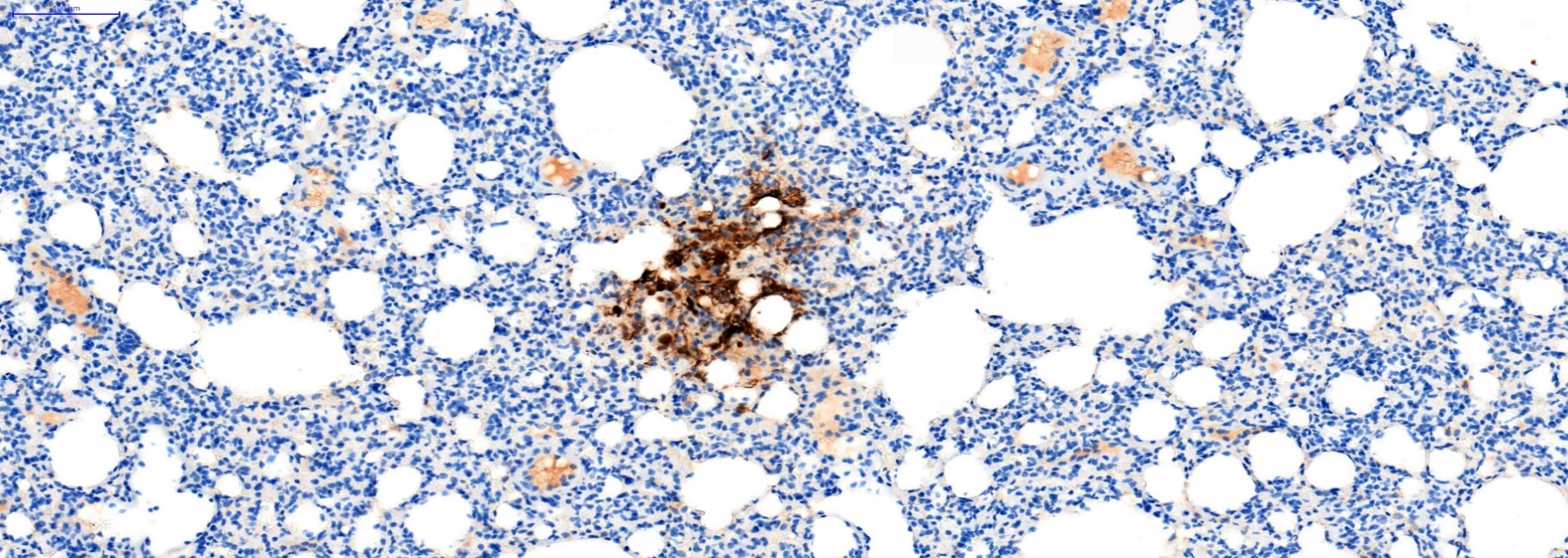

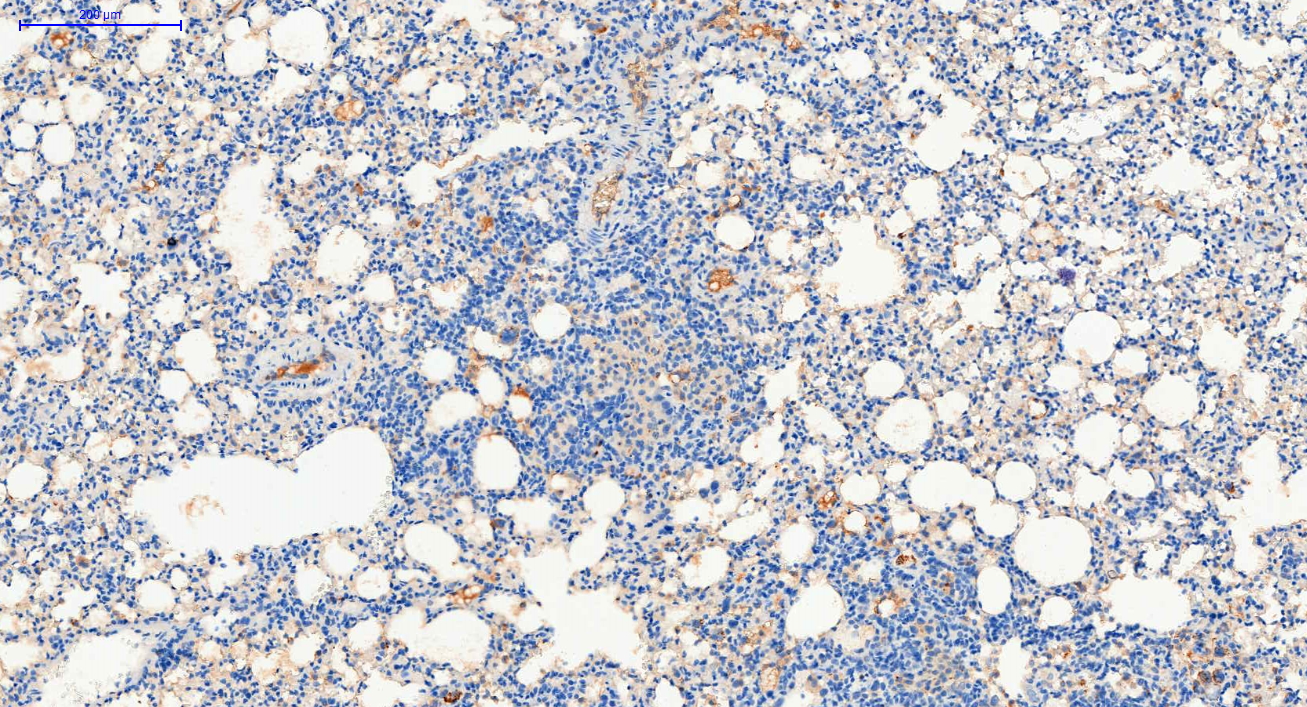

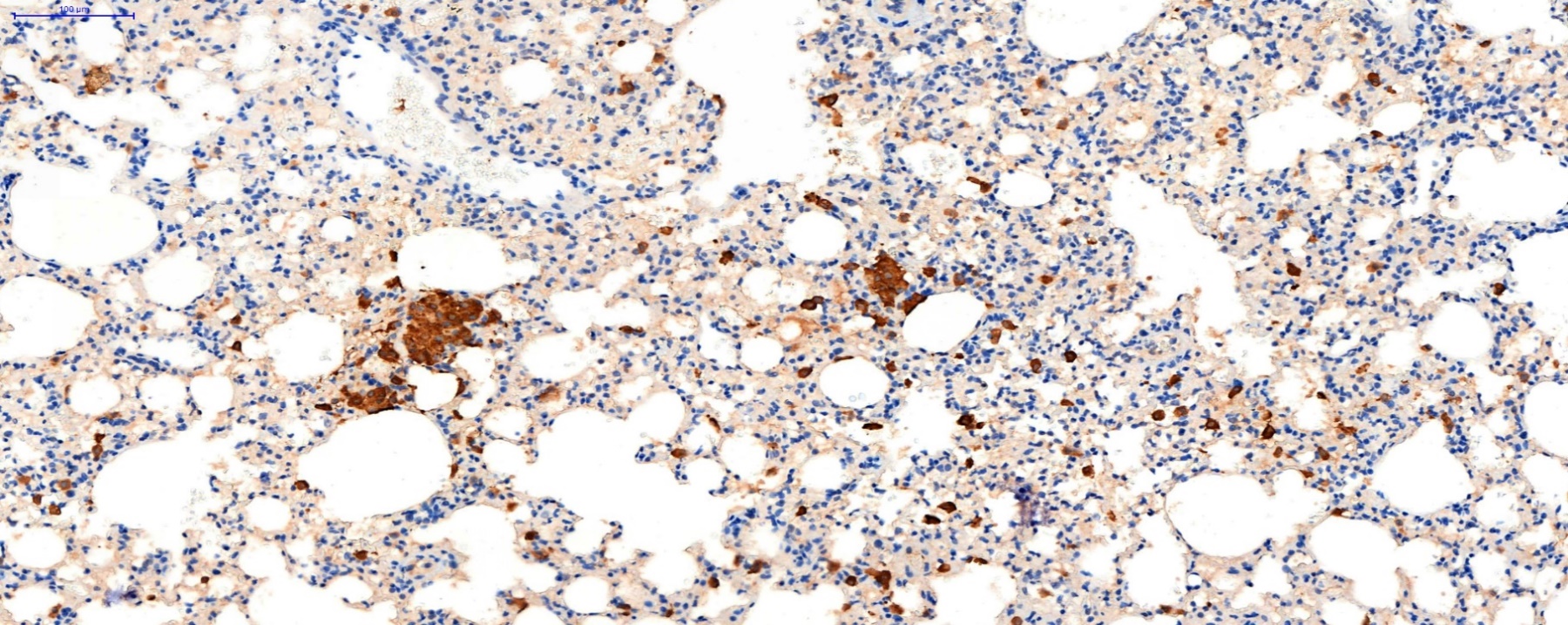

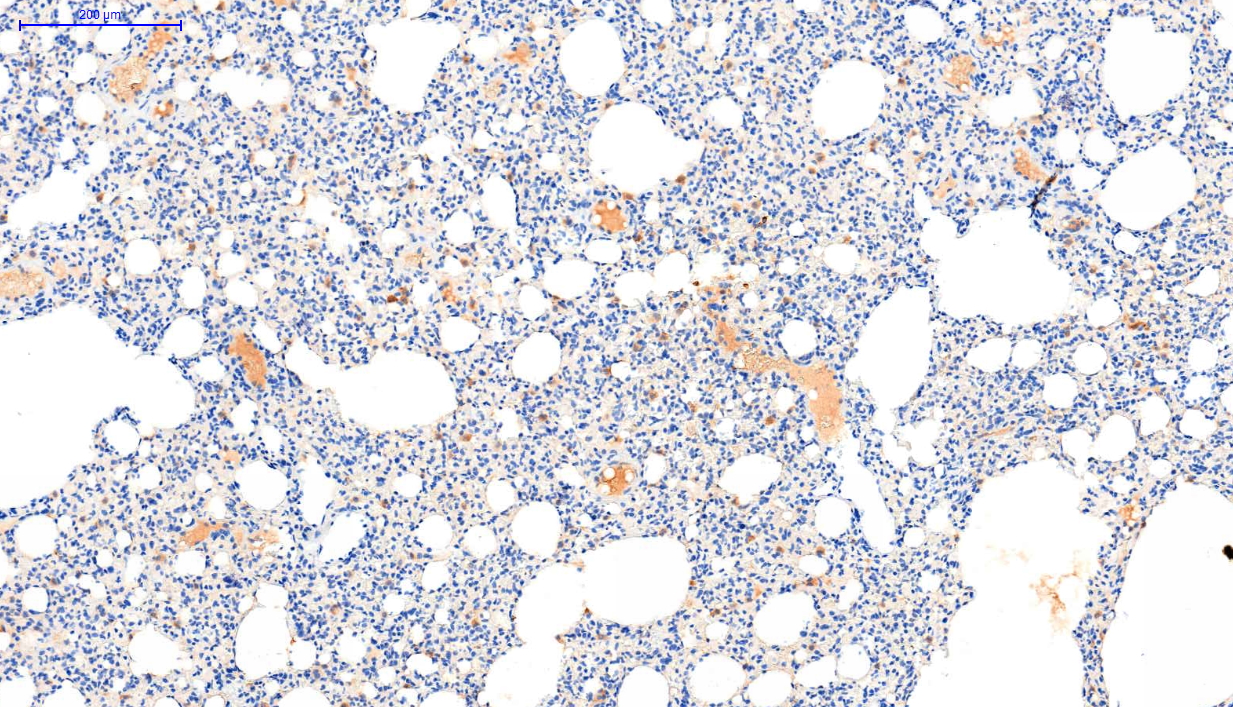

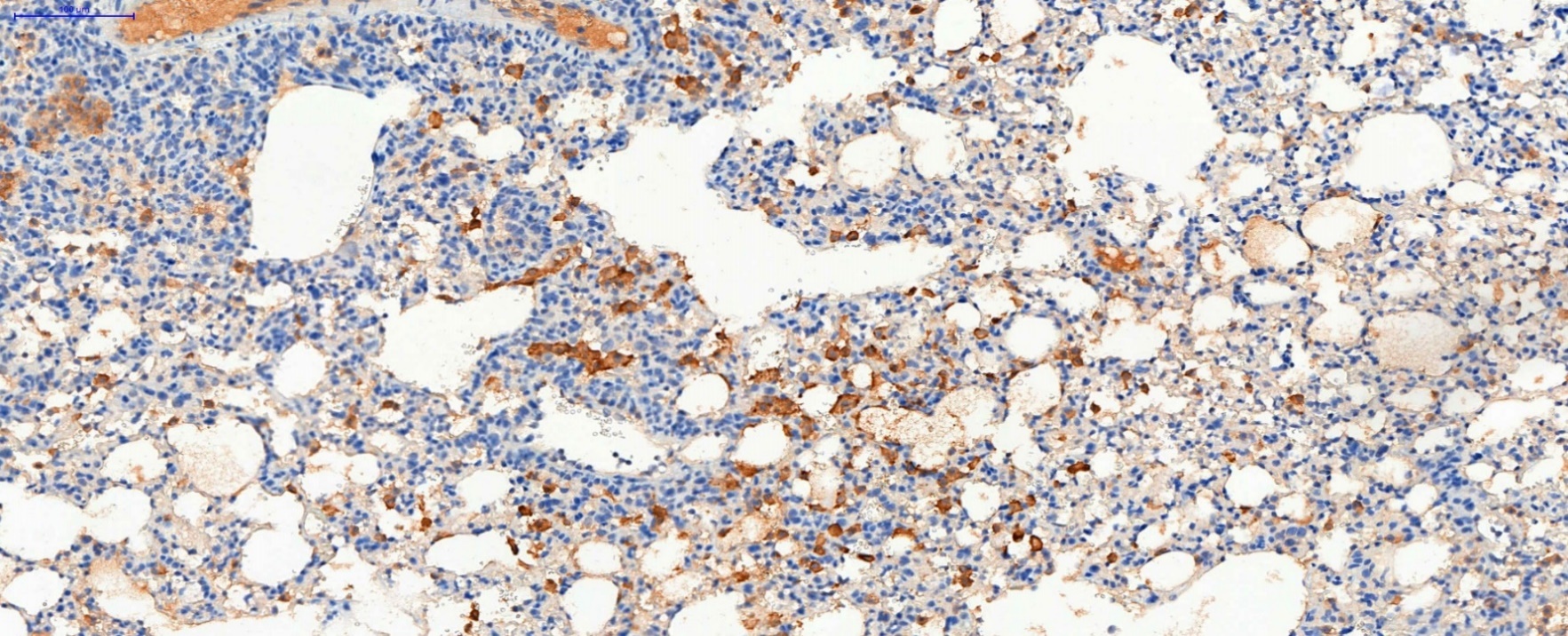


**S68**

**Day 2**

**Day 10**

**Day 14**

**S70-2**

**S66-1**


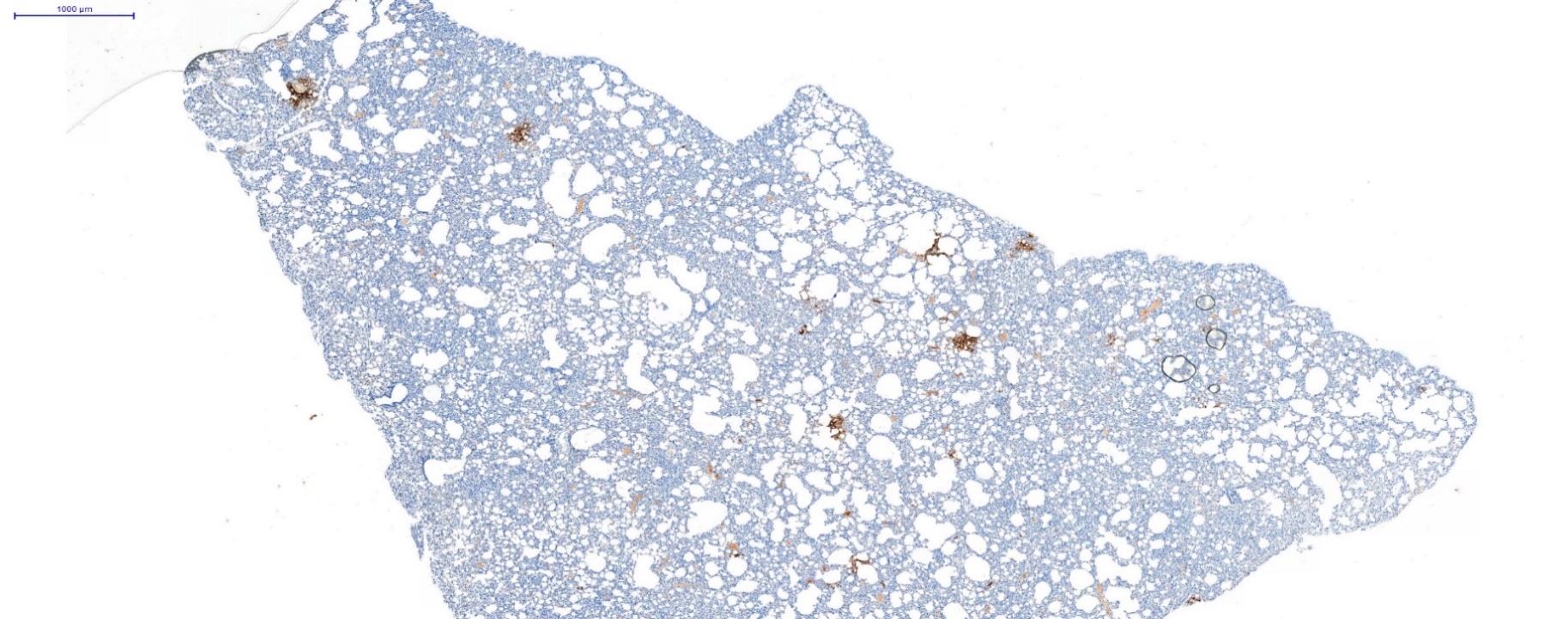

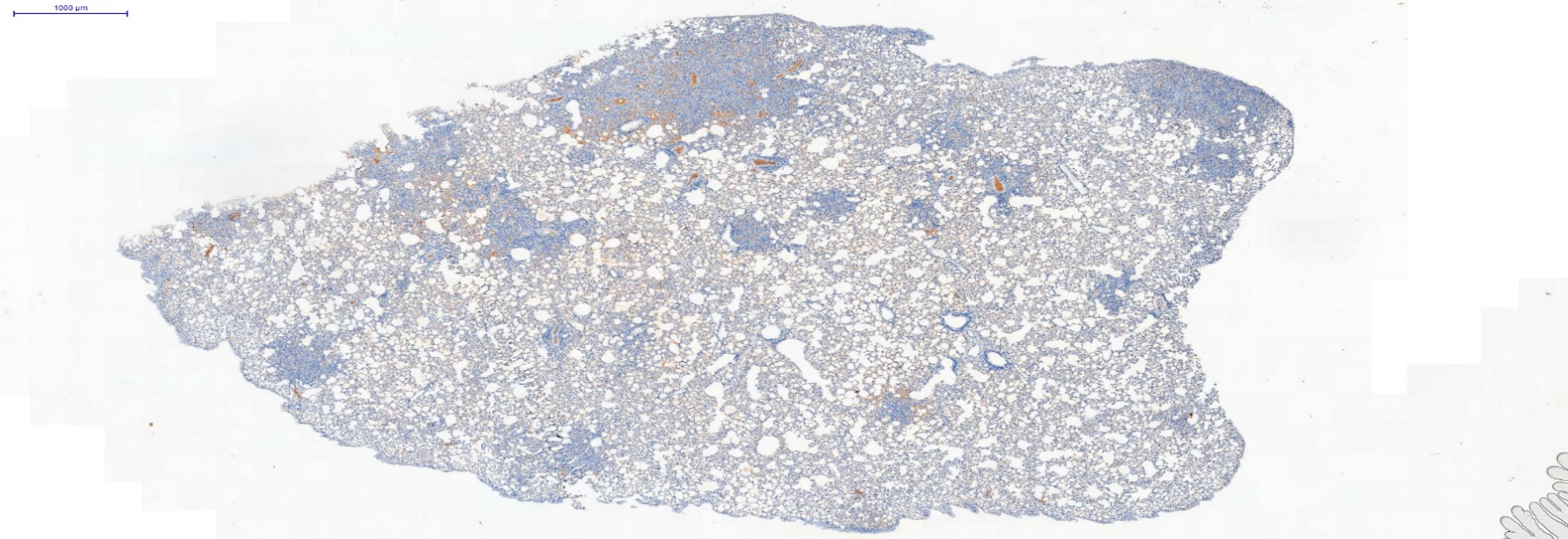


**NP**

**Spike**


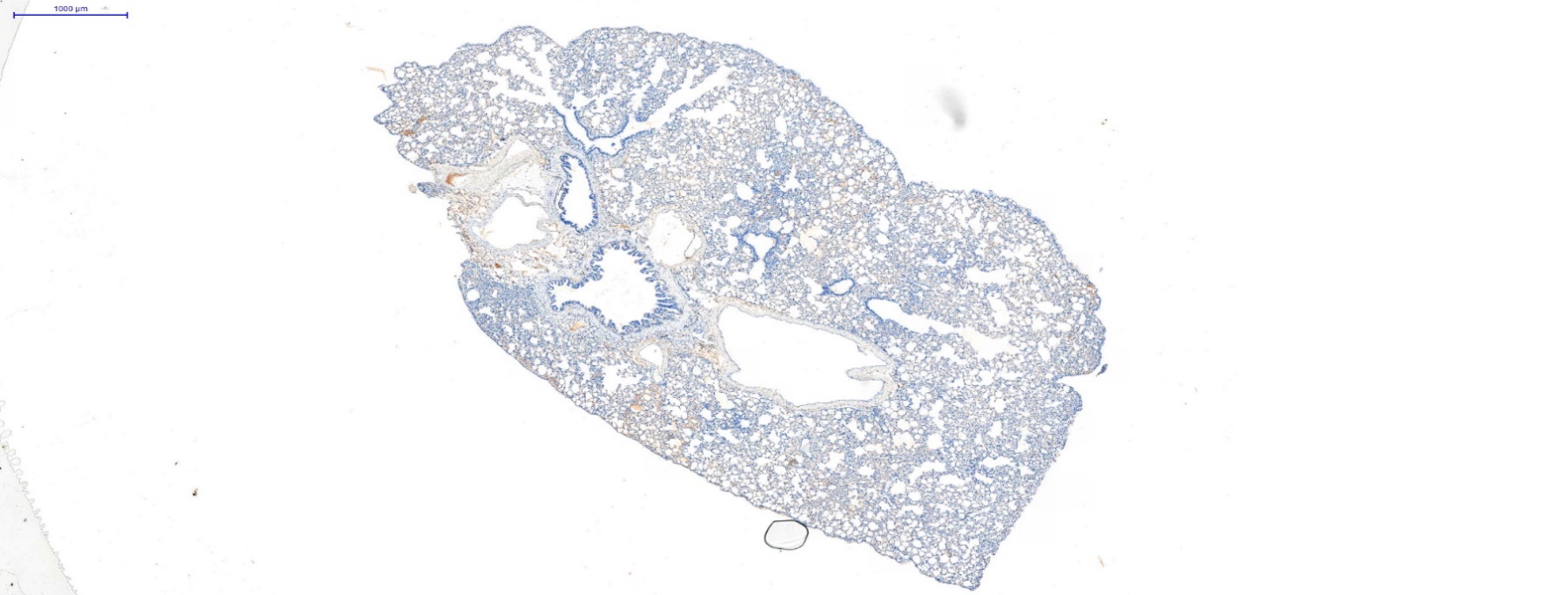

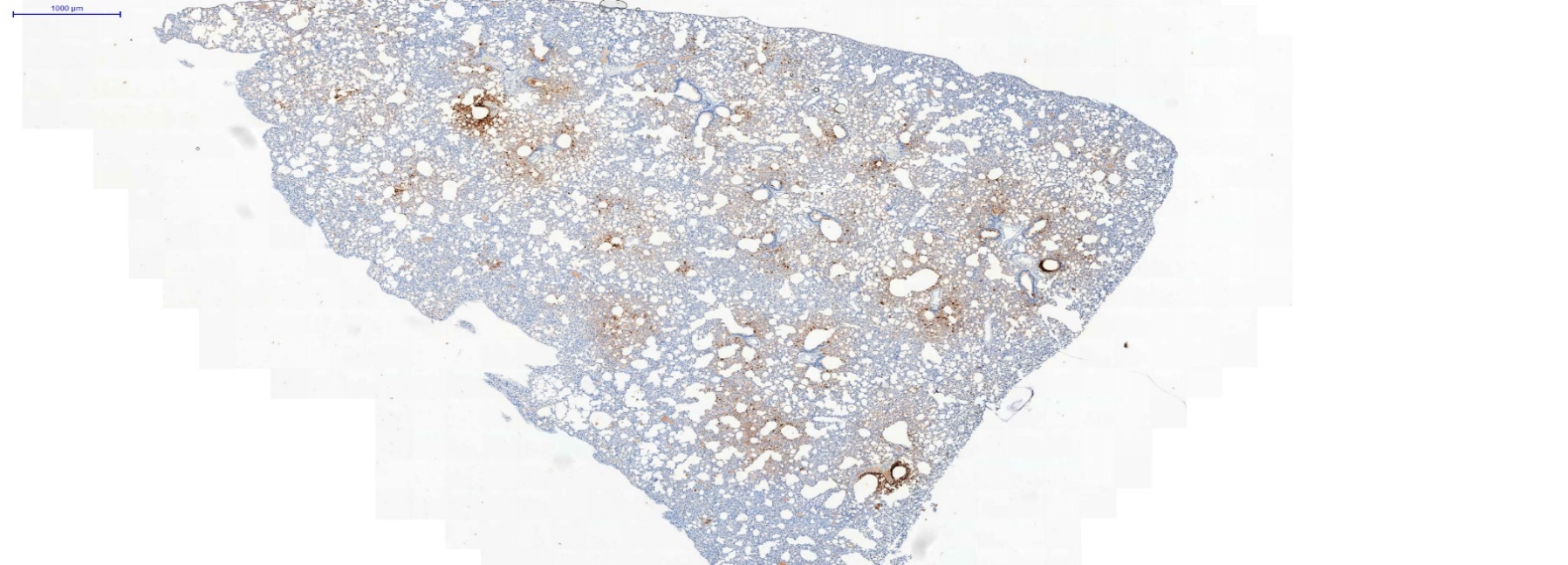


**S69 left**


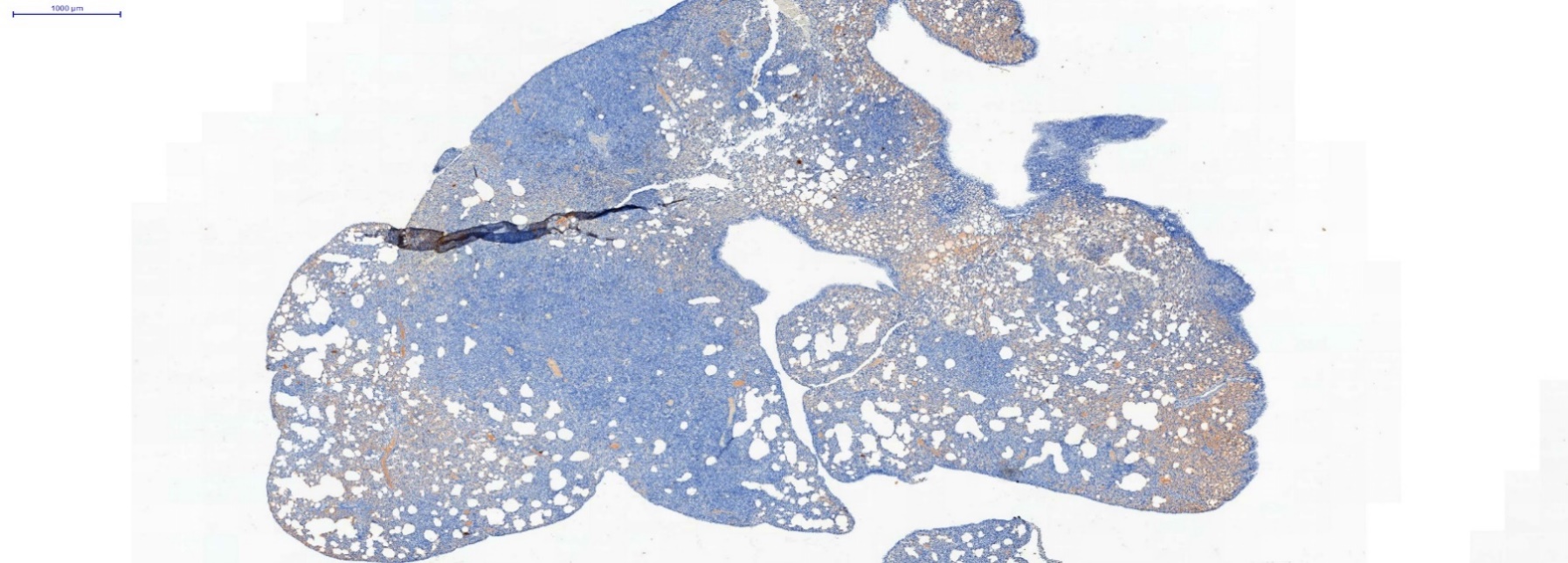


**S71 left day 7**


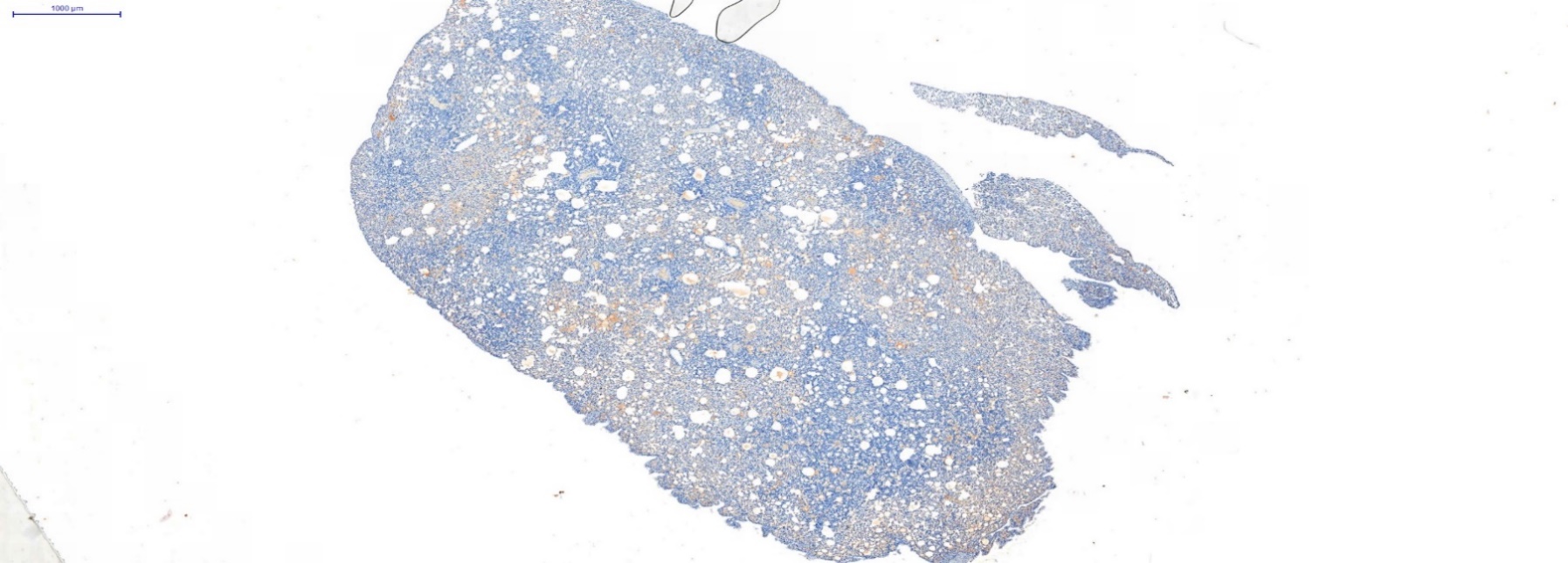


**S67 cra**

**Figure S8. Detection and localisation of SARS-CoV-2 and subgross histopathology in lung tissue in male hamsters.** Immunohistochemistry labelling of nucleoprotein (NP) and spike protein in hamster lung sections across the 2, 10 and 14 days in male hamsters infected with SARS-CoV-2/Vic-01. Sub-gross histopathology analysis of lung sections at each time point indicated. Brown labelling indicates nucleoprotein in areas of lesions taken as a focus of infection of SARS-CoV-2. Day 2**,** S69 LCau (left caudal); S71 left caudal taken at day 7 and S 67 cranial taken at day 14.


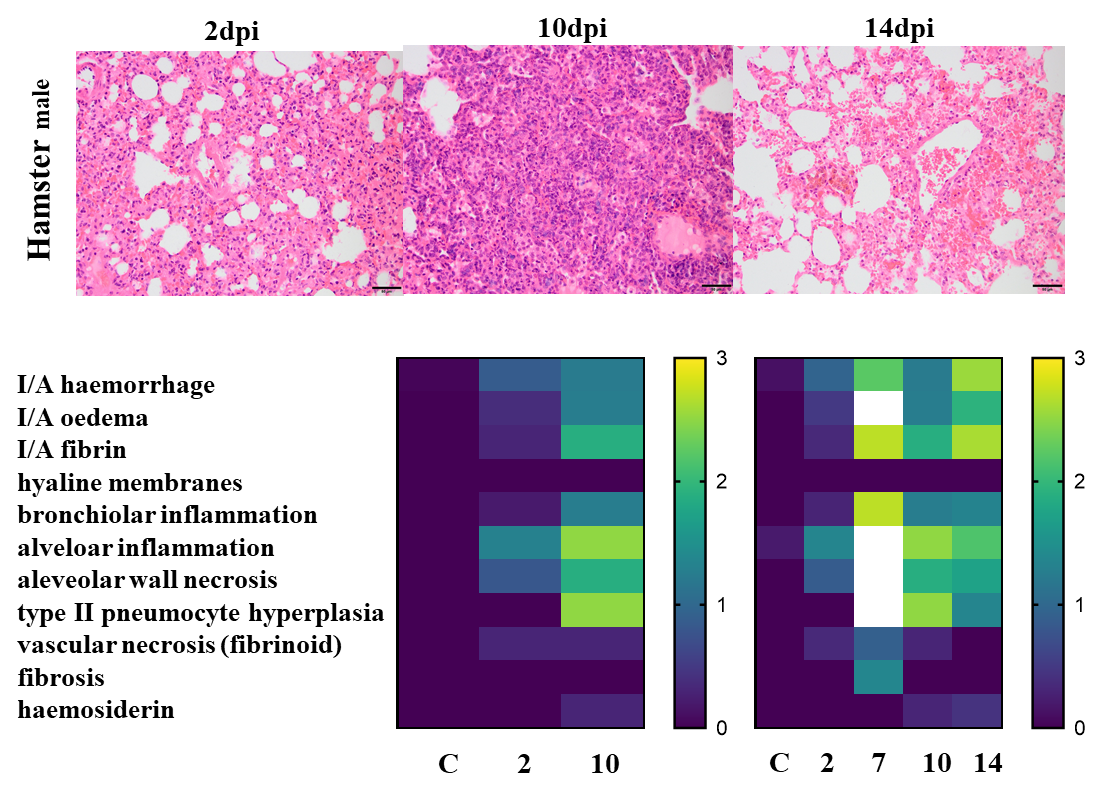


**Figure S9**. **Pathology outcomes of male hamsters.** H & E staining across the 2, 10 and 14 days post infection (dpi) in male hamsters (upper). Lower panels, heat map of histopathology comparing C (control, uninfected) with days 2 and 10 and days 2, 10 and 14. Heat map scale 0-3, white areas represent >3. Heat map graphics generated using GraphPad Prism version 9.3 software.


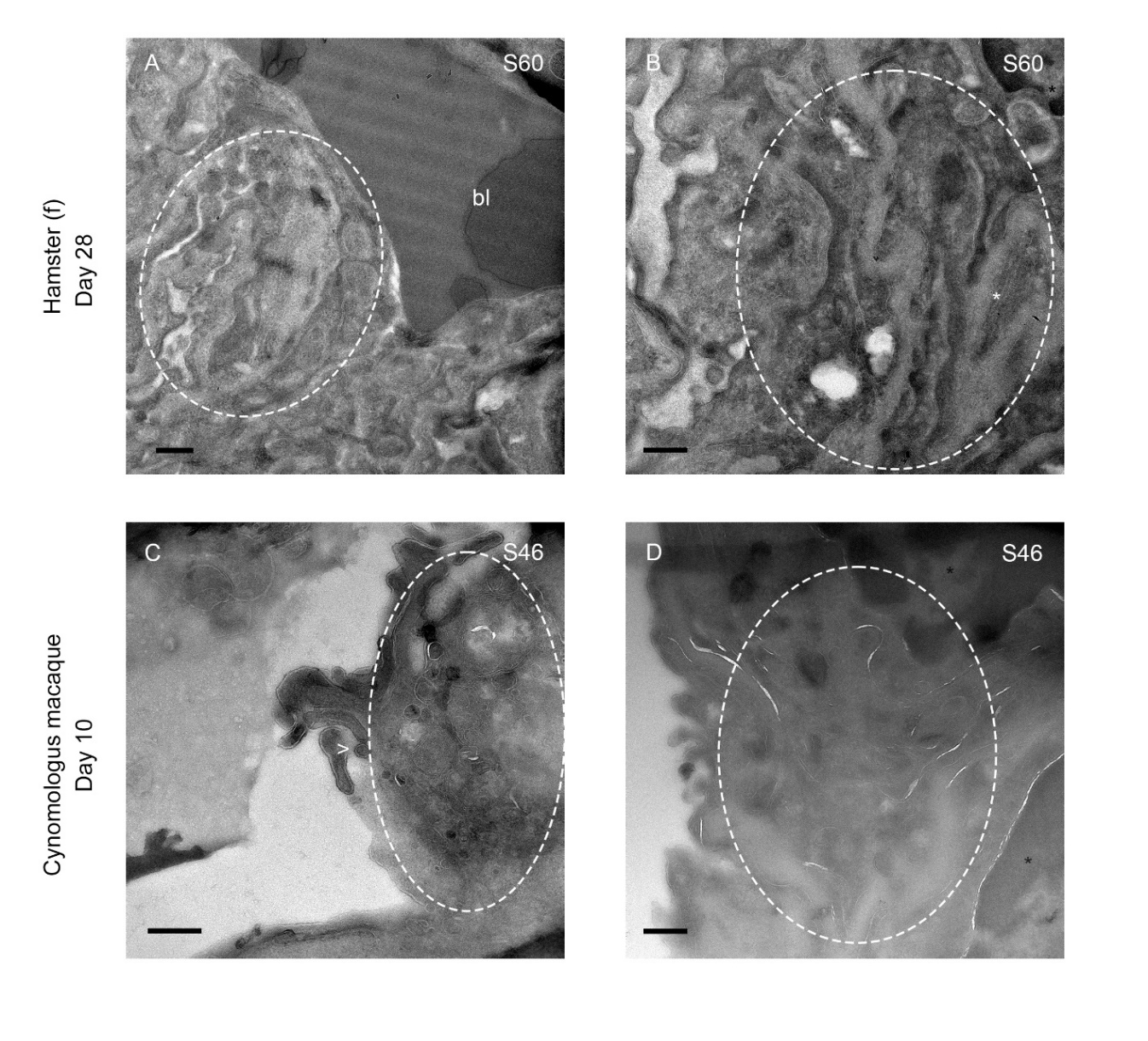


Figure S10. Electron micrographs of sections for 28 day hamster and 10 day macaque lungs. Panels A and B show disrupted cell ultrastructure as indicated in hamster S60 lungs. Images C and D indicate virions present within the ultrastructure of macaque S46 lungs at day 10 and associated disrupted cell ultrastructure. Magnifications range from 10,000x to 15,000x with all scale bars corresponding to 500nm. Annotations correspond to White open arrowheads, virions; and white dotted ellipses, regions of ultrastructure disruption, White asterisk, back asterisk and bl denote collagen fibrils, nucleus and blood respectively.


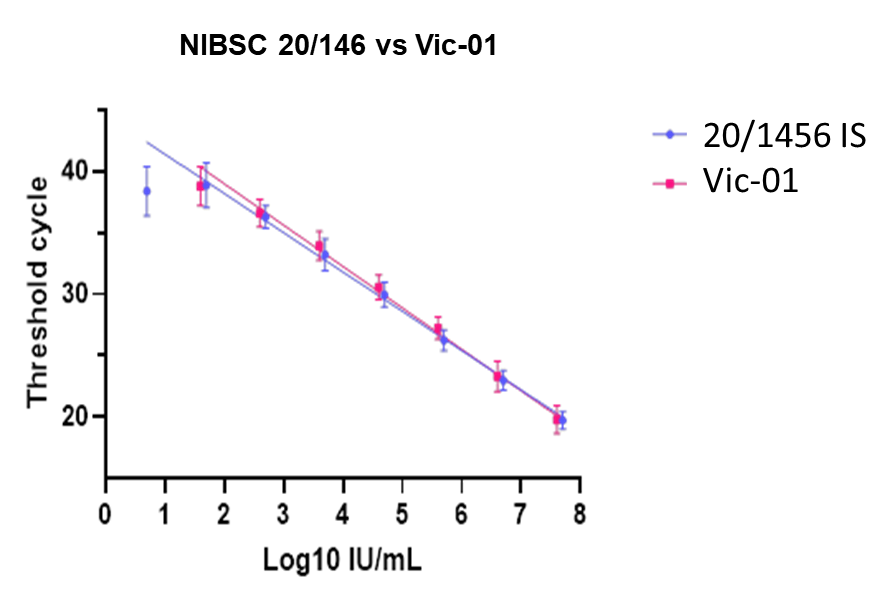


Figure S11. Regression analysis of WHO IS 20/146 and Vic-01 standard series. Triplicate analysis of NIBSC reference material 20/146 designated the WHO International Standard (IS) with a Victoria-01 (Vic-01) standard series applied in a calibration experiment. Threshold cycle data are plotted against the SARS-CoV-2 IS in International Units per mL (IU/mL).

Table S1.
